# Assessment of the Safety and Therapeutic Benefits of Convalescent Plasma in COVID-19 Treatment: A Systematic Review and Meta-Analysis

**DOI:** 10.3389/fmed.2021.660688

**Published:** 2021-04-06

**Authors:** Daniela Ferreira Barreira, Rita Adubeiro Lourenço, Rita Calisto, Daniel Moreira-Gonçalves, Lúcio Lara Santos, Paula Alexandra Videira

**Affiliations:** ^1^Department of Life Sciences, Applied Molecular Biosciences Unit, Faculdade de Ciências e Tecnologia da Universidade Nova De Lisboa, Caparica, Portugal; ^2^Portuguese Institute of Oncology Francisco Gentil, Porto, Portugal; ^3^Cancer Epidemiology Group, Portuguese Institute of Oncology Porto Research Centre (CI-IPOP), Portuguese Institute of Oncology Francisco Gentil, Porto, Portugal; ^4^Research Centre in Physical Activity, Health and Leisure, Faculdade de Desporto, Universidade do Porto, Porto, Portugal; ^5^Experimental Pathology and Therapeutics Group, Research Center, Portuguese Oncology Institute of Porto (IPO-Porto), Porto, Portugal; ^6^Surgical Oncology Department, Portuguese Oncology Institute of Porto (IPO-Porto), Porto, Portugal; ^7^Congenital Disorders of Glycosylation Professionals and Patient Associations International Network (CDG and Allies-PPAIN), Lisboa, Portugal

**Keywords:** COVID-19, convalescent plasma, meta-analysis, mortality, safety, viral clearance, clinical trials

## Abstract

**Background:** The coronavirus disease (COVID-19), caused by the Severe Acute Respiratory Syndrome Coronavirus-2 (SARS-CoV-2), prompted a global health crisis, with no available specific treatments. Convalescent plasma (CP) with neutralizing antibodies could be a promising therapeutic approach to reduce mortality.

**Objectives:** To evaluate the therapeutic potential of CP for COVID-19 and to assess its safety and efficacy in reducing the patients' mortality.

**Methods:** We retrieved clinical trial references from multiple Databases (e.g., PubMed, B-On, SCOPUS), for complete studies until November 26th 2020. We included Randomized controlled trials (RCT) and controlled non-randomized trials (CNRT), that assessed the efficacy of CP to treat hospitalized COVID-19 patients. Trials were included regardless of concomitant medications in the intervention's arms. Eleven trials met our eligibility criteria. This study was performed according to the Preferred Reporting Items for Systematic Reviews and Meta-analyses (PRISMA) guidelines. We defined a methodological protocol to extract and evaluate all pertinent baseline demographics and interventions' characteristics from trials. The primary outcomes were the safety profile of CP, measured by the type, frequency and severity of adverse events, and CP effectiveness in reducing mortality, measured by the number of deaths registered for this therapy.

**Results:** We assessed 11 trials (5 RCT and 6 CNRT) with 3,098 participants, of whom 923 patients were treated with CP. Only 32 (3.5%) of the treated patients suffered adverse events (from which 9.4% serious transfusion-related adverse events). The overall mortality rates were significantly decreased by CP administration {risk ratio (RR) 0.71, *p* = 0.005, 95% confidence interval (Cl) [0.57–0.90]}, with low heterogeneity. In the sub-analysis by period of transfusion, CP transfusion within a week of hospitalization contributed to diminished mortality rate (RR = 0.71, *p* = 0.03, 95%Cl [0.53–0.96]). CP therapy also led to significantly reduced viral loads at 72 h after transfusion (RR = 0.61, *p* = 0.04, 95%Cl [0.38–0.98]), despite high heterogeneity due to disease severity.

**Conclusion:** This meta-analysis established CP as a safe and potentially effective therapy for COVID-19, decreasing the mortality rates and promoting a swift viral clearance. Further studies are necessary to provide stronger evidence.

## Introduction

In 2020 a pandemic caused by the newly emergent disease COVID-19 has, triggered a global health crisis. The causative agent of this condition is SARS-CoV-2 (1, 2). So far, this virus has infected 82 million people worldwide and led to over 2,620 k deaths (3). The elderly population accounts for higher mortality rates, due to underlying comorbidities, such as cardiovascular or respiratory disease, diabetes, hypertension, which increase the vulnerability to this virus infection (4, 5).

Currently, there are no specific treatments for SARS-CoV-2 infection and vaccination programs with emerging vaccines have begun. Many pharmacological options are being tested in clinical trials to assess safety and efficacy in preventing COVID-19 (6, 7). Meanwhile, it is necessary to use approaches that rapidly treat COVID-19 patients and lessen the burden of this infection in the healthcare systems. A promising therapeutic option relies on the use of convalescent plasma (CP), an antibody therapy commonly employed to treat patients suffering from infections. It consists of serum collected from previously infected but now recuperated individuals (8, 9). CP is rich in neutralizing antibodies against SARS-CoV-2 and thus neutralizes the virus avoiding further infection, improving clinical outcomes (10, 11). This is particularly important when a response faster than vaccines is required and for patients with compromised immune system or rare diseases. Although promising, its efficacy and safety have been controversial due to the lack of systematic studies (12).

The goal of this review is to evaluate the potential of using CP or enriched antibodies from plasma (hyperimmune plasma) for the treatment of COVID-19 in comparison to standard treatment (ST) (treatments performed without CP), in clinical trials. Multiple aspects were assessed, such as the safety and efficacy of convalescent plasma in reducing the patients' viral load and overall mortality.

## Methods

### Eligibility Criteria

#### Types of Participants (P)

We included studies assessing severely, critically, and moderately ill hospitalized COVID-19 patients, with confirmed SARS-CoV-2 infection by qPCR, with no limitations of gender, age, or ethnicity. Patients had to be well characterized in terms of presence or absence of concomitant comorbidities, major symptoms (e.g., fever, cough, and fatigue), time onset until hospitalization and disease severity before the beginning of the treatment (severe or critically ill, and if the patients were on mechanical ventilation).

#### Types of Interventions (I)

We included interventions with convalescent plasma (without antiviral treatment) or hyperimmune plasma.

#### Comparators (C)

The CP treatment was compared with standard care or placebo. Studies where patients receive simultaneous medications (e.g., antiviral, antibiotics/antifungal, and corticosteroids) and/or respiratory support [e.g., mechanical ventilation, high-flow nasal oxygen (HFNO), low-flow nasal oxygen (LFNO)] were considered if these interventions were equally offered to both groups.

#### Types of Outcomes Measures (O)

The primary outcomes we evaluated were the safety of CP—measured by type, frequency, and severity of adverse events (grade ≥ 3)—and its effectiveness in reducing mortality. Whereas, the secondary outcomes were viral clearance, respiratory improvement, and length of hospitalization after treatment.

#### Types of Studies (S)

We considered RCT and CNRT, published in the form of abstract, full-text article or data published in trial registries, written in English, Portuguese or Spanish.

### Information Sources

The following electronic databases were searched: PubMed/MEDLINE (2020–2020), Web of Science (2020–2020), B-on (2020–2020), EBSCO (2020–2020) and SCOPUS/EMBASE (2020–2020), as well as clinical trials databases such as Clinicaltrials.gov. In addition, we manually searched studies by screening the reference list of relevant publications on the topic. The last search was performed on November 26th 2020.

### Search

The information was obtained from the databases and trial registers by searching the following keywords/terms.

#### PubMed

(COVID-19 [Supplementary Concept] OR “COVID-19”[All Fields] OR “severe acute respiratory syndrome coronavirus 2”[All Fields] OR “2019-nCoV”[All Fields] OR “SARS-CoV-2”[All Fields] OR ((“Wuhan”[All Fields] AND (“coronavirus”[MeSH Terms] OR “coronavirus”[All Fields])) AND 2020[All Fields])) AND (COVID-19 serotherapy[MeSH Terms] OR “COVID-19 serotherapy”[All Fields] OR COVID-19 serotherapy[Text Word] OR “Plasma immunoglobulins”[All Fields] OR “immune-globulin” [All Fields] OR “Plasma immunoglobulins”[All Fields] OR “hyper-immune” [All Fields] OR “Hyperimmune plasma”[All Fields] OR “Convalescent Plasma”[All Fields]).

#### Clinicaltrials.gov

Condition or disease: COVID-19.

Intervention/treatment: COVID-19 serotherapy OR Plasma immunoglobulins OR immune-globulin OR Plasma immunoglobulins OR hyper-immune OR Hyperimmune plasma OR Convalescent Plasma.

#### SCOPUS

COVID-19 OR severe acute respiratory syndrome coronavirus 2 OR 2019-nCoV OR SARS-CoV-2 OR Wuhan AND COVID-19 serotherapy OR Plasma immunoglobulins OR immune-globulin OR Plasma immunoglobulins OR hyper-immune OR Hyperimmune plasma OR Convalescent Plasma.

#### EBSCO

TX: COVID-19 OR severe acute respiratory syndrome coronavirus 2 OR severe acute respiratory syndrome coronavirus 2 OR 2019-nCoV OR SARS-CoV-2 OR Wuhan.

AND

TX: COVID-19 serotherapy OR Plasma immunoglobulins OR immune-globulin OR Plasma immunoglobulins OR hyper-immune OR Hyperimmune plasma OR Convalescent Plasma.

#### B-on

TX: COVID-19 OR severe acute respiratory syndrome coronavirus 2 OR 2019-nCoV OR SARS-CoV-2 OR Wuhan.

AND

TX: COVID-19 serotherapy OR Plasma immunoglobulins OR immune-globulin OR Plasma immunoglobulins OR hyper-immune OR Hyperimmune plasma OR Convalescent Plasma.

#### Web of Science

Title/keywords/Abstract: COVID-19 OR severe acute respiratory syndrome coronavirus 2 OR 2019-nCoV OR SARS-CoV-2 OR Wuhan.

AND

Title/keywords/Abstract: COVID-19 serotherapy OR Plasma immunoglobulins OR immune-globulin OR Plasma immunoglobulins OR hyper-immune OR Hyperimmune plasma OR Convalescent Plasma.

### Study Selection

**Figure 1:**
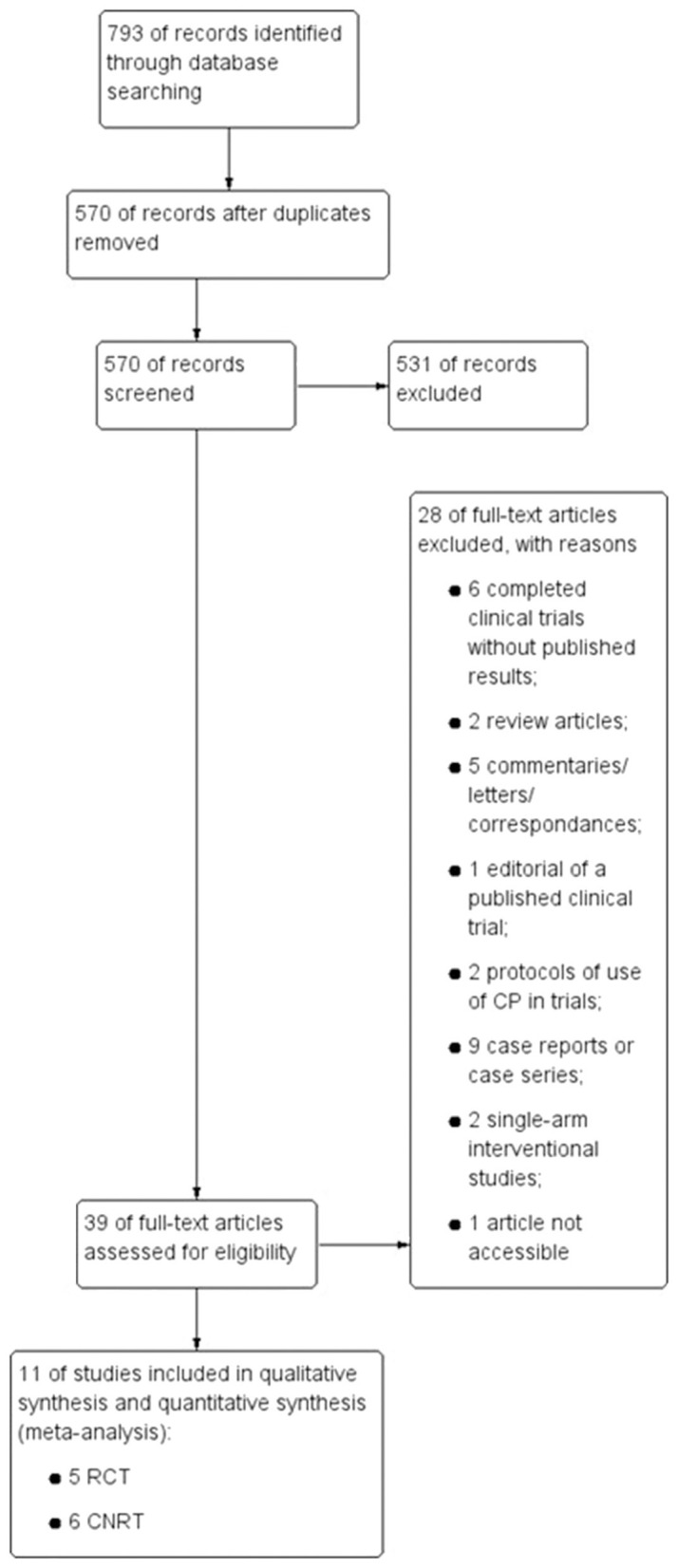
PRISMA Flow diagram illustrating the study selection process. Schematic illustration indicating the various steps from the Database search until the inclusion of the selected trials for this meta-analysis. The number of trials excluded, and the reason for exclusion are also described in this scheme. RCT, Randomized Clinical trials; CNRT, Controlled Non-Randomized Trials.

Two independent reviewers first screened the title and abstract of all the retrieved records to avoid including duplicated publications and to select potential studies for further assessment of eligibility. A PRISMA flow diagram illustrates the study selection process, including the number of retrieved studies, the number and rationale for included and excluded references (Figure 1 and Supplementary Table 1).

### Data Collection and Management

From the eligible studies, we extracted the information deemed relevant, such as patient characteristics (age, gender, main symptoms, disease onset, and severity), type of intervention and comparators, as well as expected outcomes/outcomes measures and risk of bias. One author was responsible for collecting information and a second reviewer confirmed the extracted data. All disagreements/doubts were resolved through the input of all authors.

### Risk of Bias in Individual Studies

The bias assessment of the included studies was performed by two independent review authors and a third author verified the accuracy and helped achieve consensus for a final decision in case of disagreement. This assessment was performed at the study and primary outcome level. For RCT we used Risk of Bias 2.0 (RoB 2) tool and for non-RCT we used the Risk of Bias in Non-randomized Studies—of Interventions (ROBINS-I) tool, as described in the Cochrane Handbook for Systematic Reviews of Interventions. Considering these aspects, the risk of bias for each domain was judged as “Low,” “High,” or “Some concerns” risk for RoB 2 tool and “Low,” “Moderate,” “Serious,” or “Critical” risk for ROBINS-I tool.

As a result, presented a summary figure indicating the judgement of each domain for all references.

### Summary Measures

To conduct meta-analysis to the selected controlled trials we considered dichotomous, continuous, and time-to-event data. The dichotomous data was assessed by the risk ratio (RR) with 95% Confidence interval (CI), after collecting the total number of patients in each intervention arm and the number of events. For the continuous data evaluation, we extracted the means, standard deviation, and total number of participants in each intervention group. With these values we calculate the mean difference (MD) with 95% CI between the intervention arms of all studies together, since the outcome measurements are the same throughout the studies.

### Synthesis of Results

Data synthesis was performed according to recommendations in the Cochrane Handbook for Systematic Reviews of Interventions (13), using Review Manager 5.5 (software Cochrane Collaboration). The random-effects mode was used because we assumed that the true effect size varies from one study to the next, and that the studies in our analysis represent a random sample of effect sizes that could have been observed. For binary outcomes, we estimated between-study variance with the Mantel–Haenszel method. The inverse variance method was used for continuous outcomes or outcomes where HRs were available.

To appraise the clinical heterogeneity among the trials' treatment effect, considering possible variabilities in the trials' conditions, we used the CHI^2^ test with a significance level of 0.1, and the *I*^2^ statistic (*I*^2^ classification: >30%—moderate heterogeneity; >75%—considerable heterogeneity). The *I*^2^ statistic quantifies inconsistencies throughout the studies, assessing its impact on the meta-analysis, without depending on the number of studies. Therefore, when we obtained high levels of heterogeneity (*I*^2^ > 75%) we attempted to determine possible sources through sub-group analysis.

## Results

We conducted a wide Database search and identified 11 studies fitting our criteria—five RCTs and six CNRTs—which were included for qualitative and quantitative evaluation, as indicated in Figure 1. Nine studies assessed the efficacy of CP for treating patients with severe or life-threatening COVID-19 (14–21), while two studies evaluated the potential of CP in moderately ill hospitalized patients (22, 23). Altogether, 923 patients were submitted to CP treatment of a total population of 3,098 patients (2,553 critical or severely ill patients and 545 moderately ill patients).

All studies consisted of two intervention groups, the CP and the control/ST group. Regardless of the intervention group, all patients received concomitant medications, for instance, antivirals, antibiotics, or traditional Chinese medication, as recommended by standard care protocols to ensure their well-being. Detailed methodology, baseline characteristics, interventions characteristics, and the major results of these studies are provided in Supplementary Tables 2–5, respectively.

### Qualitative Analysis—Risk of Bias of the Included Studies

Briefly, three RCT were appraised at low risk of bias (12, 22, 23) whereas two presented some bias concerns (16, 18), especially in the allocation concealment. The CNRT were evaluated at an overall serious or critical risk of bias, mostly due to confounders. Regarding the bias assessment across studies, no selective reporting was observed. Although in some outcomes only a few trials were eligible for meta-analysis, this does not constitute a risk of bias because the remaining trials did not propose to assess such outcomes. The full bias analysis of the RCT and CNRT is summarized in Supplementary Tables 6, 7, respectively.

### Quantitative Analysis of the Studies' Outcomes

#### Primary Outcome: Mortality

**Figure 2:**
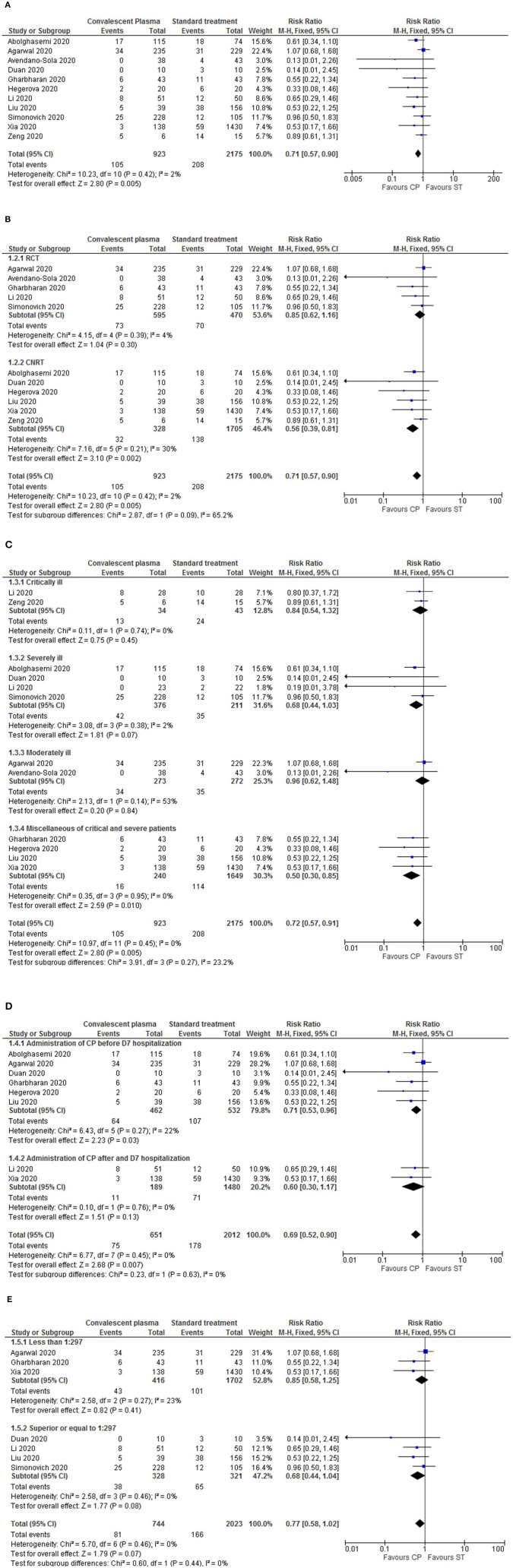
Forest plots for mortality, and the respective sub-analysis, following CP treatment. **(A)** For the overall mortality, the results showed a significantly decreased mortality rate in the CP intervention group in comparison to standard treatment. The overall heterogeneity levels are low; therefore, the results for this outcome reported are consistent. Nonetheless, a sensitivity analysis was performed by multiple sub-analysis. **(B)** Sub-analysis by study design (RCTs vs. CNRTs). **(C)** Sub-analysis by disease severity (critically, severely, moderately ill and miscallaneous of critical and severe patients). **(D)** Sub-analysis by administration period of CP (before or after 7 days of hospitalization). **(E)** Sub-analysis by antibody titers administered (<1:297 or ≥1:297). Different sizes of data markers correspond to the relative weight assigned in the pooled analysis. Diamond marker indicates the overall result.

We first assessed the general mortality for all studies (12, 14–23). As shown in Figure 2A, the administration of CP significantly reduced the mortality rates in comparison to ST (RR = 0.71, *p* = 0.005, 95%Cl [0.57–0.90], 3,098 patients total), with low heterogeneity levels (*p* = 0.42, *I*^2^ = 2%). No specific time point was evaluated since the time frames measured differed among studies or were not reported.

Next, we performed sensitivity assay through sub-analysis of the mortality outcome by type of study design (RCT vs. CNRT), period of CP administration (before or after 7 days of hospitalization), disease severity (critical, severe, or moderate) and antibody titers administered to patients [higher or lower than the mean value (1:297)].

All sub-analysis demonstrated a tendency for decreased mortality rates in the CP intervention group. Furthermore, when sub-analyzing by study design, there was a significant decrease of mortality in the CNRTs (RR = 0.56, *p* = 0.002, 95%Cl [0.39–0.81], 2,033 patients) (Figure 2B). By disease severity, the diminished mortality was significantly lower in the miscellaneous of critical and severe patients (RR = 0.50, *p* = 0.010, 95%Cl [0.30–0.80]) (Figure 2C). It was noted diminished mortality in the sub-group of patients transfused with CP within a week of hospitalization (RR = 0.71, *p* = 0.03, 95%Cl [0.53–0.96], 994 patients; Figure 2D). When sub-analyzing by antibody titers, no significant differences related to mortality were observed (Figure 2E).

In the sub-analysis by study design, the heterogeneity within the intra-groups was low but overall moderated (*p* = 0.09, *I*^2^ = 65.2%), indicating a variation in the results' reporting from RCT to CNRT (Figure 2B). Contrary, the sub-analysis by disease severity had moderate heterogeneity in the moderately ill sub-group (*p* = 0.14, *I*^2^ = 53%), but a low overall heterogeneity (Figure 2C). All other sub-analysis (including specific sub-groups) had low heterogeneity levels.

#### Primary Outcome: Safety

Regarding the safety of the CP therapy, 7 studies in this review reported patients suffering from adverse effects (12, 14, 15, 18, 20, 22, 23), accounting for a total of 32 patients in a population of 923 patients (3.5%) receiving CP. Among the 32 patients that suffered adverse events, 3 experienced severe adverse effects (9.4%), whereas 29 presented non-severe adverse effects. The most severe adverse events were transfusion-associated adverse events (grade 3 or 4). Yet, all patients recovered without sequelae. The non-severe reactions were mostly minor allergic reactions or non-hemolytic febrile reactions resulting from the transfusion.

#### Secondary Outcome: Viral Clearance

**Figure 3:**
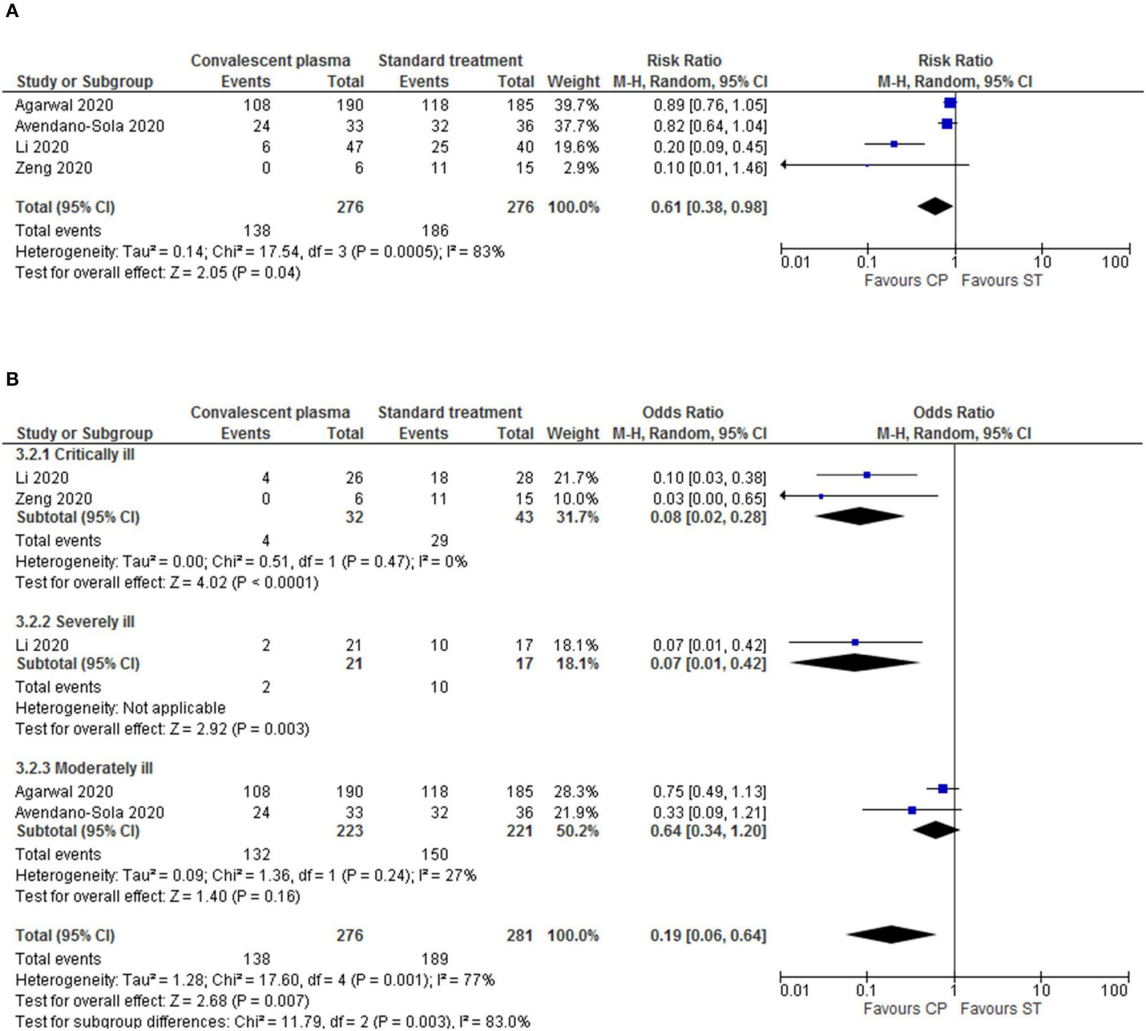
Forest plots depicting viral clearance at 72 h after CP transfusion, and respective sub-analysis. **(A)** At 72 h after CP administration, the results showed a significantly reduced number of positive laboratory-confirmed SARS-CoV-2 infection in the CP intervention group. Nevertheless, the overall heterogeneity levels are high; therefore, we conducted a sub-analysis. **(B)** Sub-analysis by disease severity (Critical, Severe, and Moderate clinical status). Different sizes of data markers correspond to the relative weight assigned in the pooled analysis. Diamond marker indicates the overall result.

The patients' viral clearance was quantitatively assessed at 72 h post-treatment for 4 studies (18, 21–23). As shown in Figure 3A, CP therapy leads to a significant decrease in the viral load within 72 h (RR = 0.61, *p* = 0.04, 95%Cl [0.38–0.98], 552 patients). Since the data had a high heterogeneity (*p* = 0.0005, *I*^2^ = 83%), we proceed to a sub-analysis of this outcome by disease severity (Figure 3B). The result showed that in the critical sub-group, the viral load at 72 h is greatly reduced in CP compared to ST (RR = 0.08, *p* < 0.0001, 95%Cl [0.02–0.28], 75 patients). In the moderate sub-group, despite a tendency for reduced viral load in the CP group, there is no statistical difference (RR = 0.64, *p* = 0.16, 95%Cl [0.34–1.20], 444 patients). We could not withdraw any relevant information from the severe sub-group with only one trial. The heterogeneity of the other sub-groups was generally low, but with variation across sub-groups (*p* = 0.003, *I*^2^ = 83%), hinting that disease severity is a relevant variable in the reported data for the viral clearance outcome (Figure 3B).

#### Secondary Outcome: Length of Hospitalization

**Figure 4:**
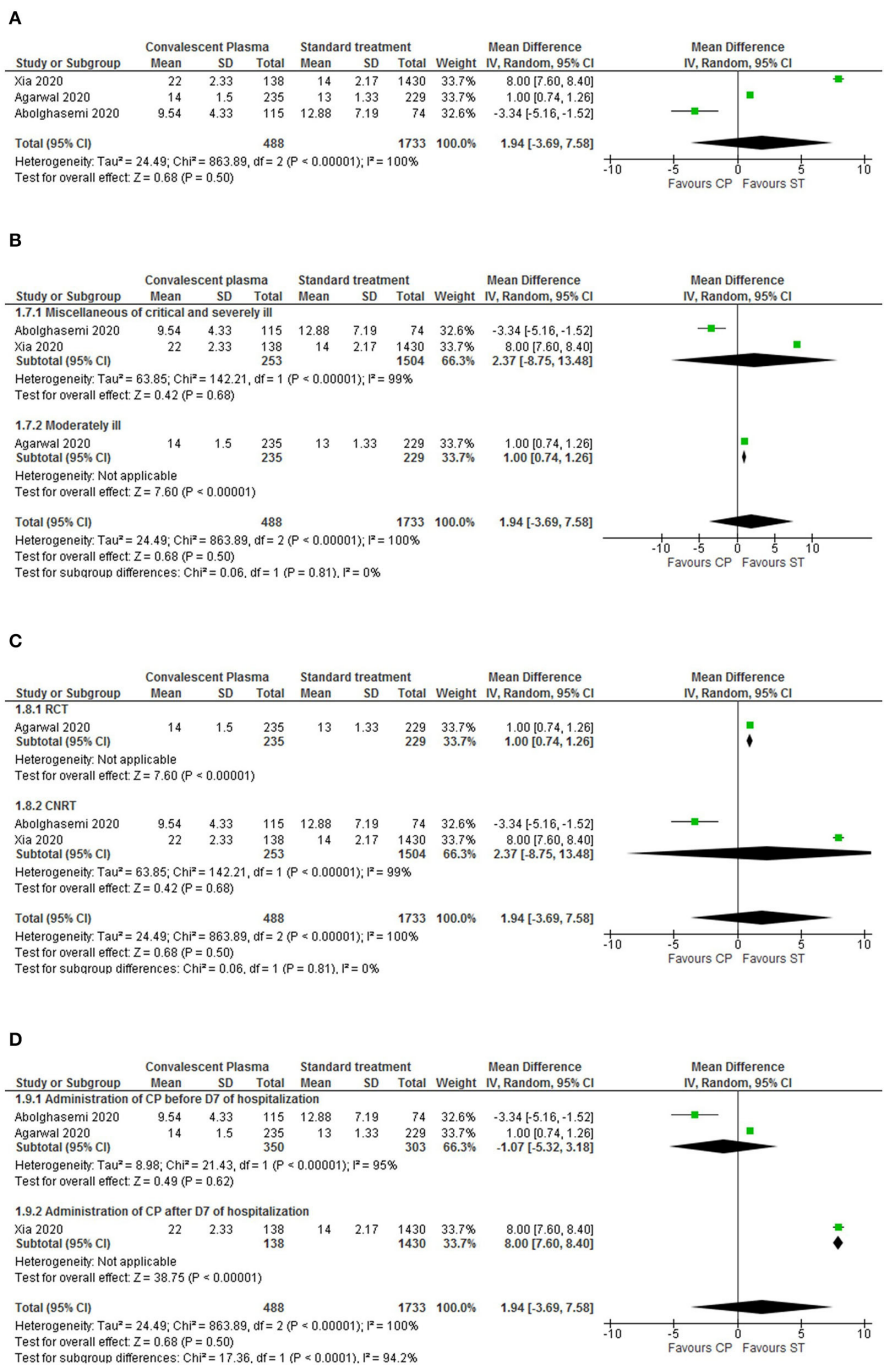
Forest plots for length of hospitalization analysis and sub-analysis assessing CP intervention and standard treatment. **(A)** Beginning at hospital admission, the hospitalization length results showed a significantly decreased period of hospital stay for the standard treatment. However, the overall heterogeneity levels are high; therefore, we conducted a sub-analysis. **(B)** Sub-analysis by disease severity (miscellaneous of critical and severe patients, and moderately ill patients). **(C)** Sub-analysis by study design (RCTs vs. CNRTs). **(D)** Sub-analysis by administration period of CP (before or after 7 days of hospitalization). The results are presented in mean and standard deviation (SD). Different sizes of data markers correspond to the relative weight assigned in the pooled analysis. Diamond marker indicates the overall result.

We then assessed the impact of CP or ST into the length of hospitalization. Here, we only considered the studies in which it was possible to present the mean length of hospitalization, evaluating the period since hospital admission (14, 20, 22) or CP treatment beginning until discharge (12, 23). For the periods starting with hospital admission, the results indicated that the control group has a tendency for reduced hospitalization in comparison to the CP group, but not statistically significant (Figure 4A). Since the data presented high heterogeneity (*p* < 0.00001, *I*^2^ = 100%), we sub-analyzed the length of hospitalization by disease severity, study design and moment of CP administration (before or after 7 days of hospitalization). All sub-analysis also presented a tendency, but not significant, for diminished hospitalization for ST (Figures 4B–D). In addition, sub-groups were either highly heterogeneous or had only one trial. This high heterogeneity indicates that the length of hospitalization was highly variable within sub-groups or not all patients respond in the same way.

Regarding the evaluation of the length of hospitalization since CP treatment beginning, the results also showed a non-significant tendency favoring ST (Supplementary Figure 1). Furthermore, to assess if the mortality outcome had an impact on the hospitalization period, we evaluated the mortality rate specifically of the trials involved in this outcome (Supplementary Figures 2A,B). The results, although not significant, showed a tendency favoring the CP treatment.

#### Secondary Outcome: Respiratory Improvement

The clinical improvement was an outcome measured by the need for oxygen supplementation after CP transfusion. Therefore, we evaluated the need for invasive (12, 14, 17, 22, 23) and non-invasive (12, 22, 23) mechanical ventilation after treatment and until termination of the studies' follow-up.

**Figure 5:**
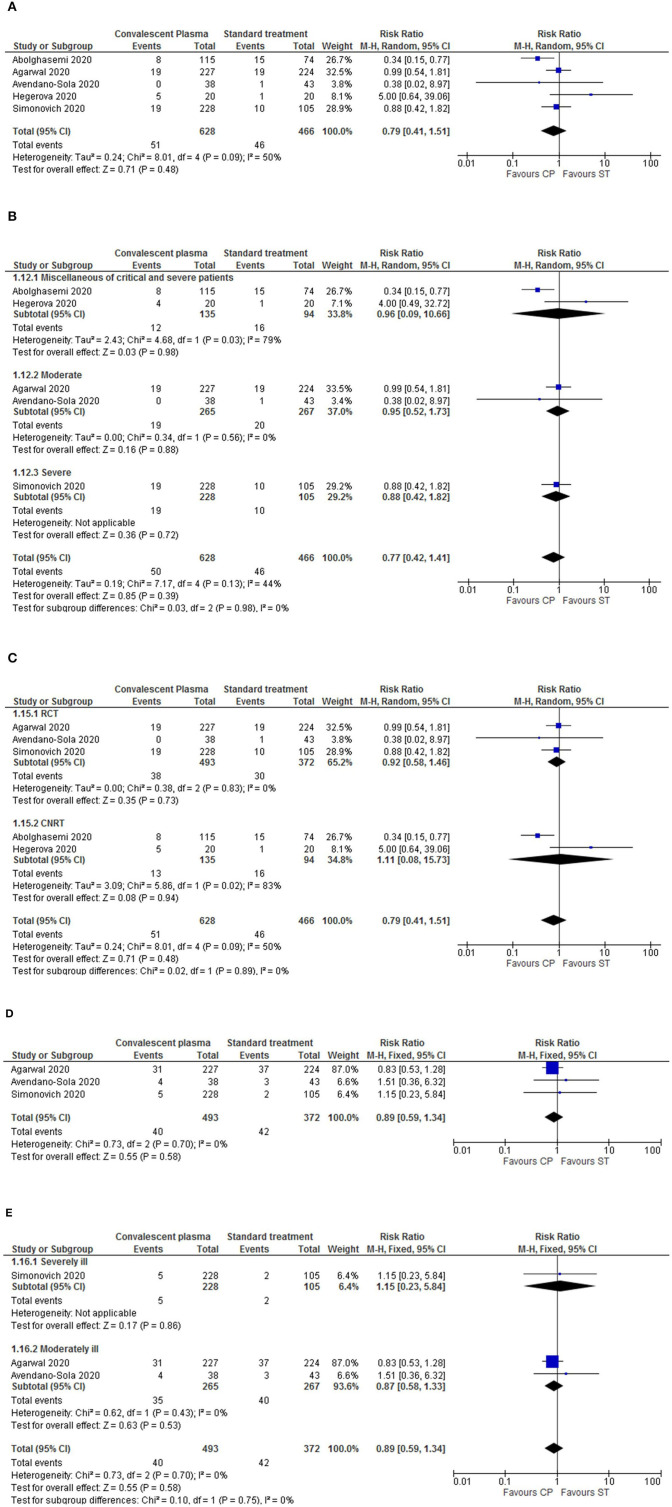
Forest plots assessing the need for invasive **(A–C)** and non-invasive **(D,E)** mechanical ventilation in CP intervention and standard treatment. **(A)** Concerning the need of invasive mechanical ventilation both groups showed no difference although a tendency favoring the CP treatment can be observed. The results also indicate moderate heterogeneity levels; therefore we conducted a sub-analysis. **(B)** Sub-analysis by disease severity (miscellaneous of critical and severe patients, and moderately ill patients). **(C)** Sub-analysis by study design (RCT vs. CNRT). **(D)** The analysis regarding the need of non-invasive mechanical ventilation showed that both groups presented no significant difference, although patients in the CP group had lower necessity of supplemental oxygen. The heterogeneity levels were null, thus a sensibility analysis by disease severity was performed **(E)**. Different sizes of data markers correspond to the relative weight assigned in the pooled analysis. Diamond marker indicates the overall result.

According to the analysis of patients' requirement of invasive mechanical ventilation (Figure 5A), the results showed no difference between CP and control groups, although it hints for less mechanical ventilated patients in the CP group. Notwithstanding, the heterogeneity analysis indicates a moderate level of variation across studies (*p* = 0.09, *I*^2^ = 50%). As such, we proceeded for sub-analysis by disease severity and by study design that demonstrated no significant difference for either sub-groups, though CP treatment tended to lessen the need for mechanical ventilation (Figures 5B,C). Despite the high heterogeneity of the severe/critical sub-group (*p* = 0.03, *I*^2^ = 79%), there was no heterogeneity across sub-groups, indicating that the overall results concerning oxygen supplementation are consistent.

Regarding the need for non-invasive mechanical ventilation, the results also did not present a difference between intervention groups, but there is clearly a tendency favoring CP group (Figure 5D). Since this result had a null heterogeneity level, we performed a sensitivity analysis by disease severity, which revealed a consistency in the presentation of the data by the trials with a tendency favoring fewer oxygen requirement in the CP group in moderately ill patients (Figure 5E).

## Discussion

Despite being historically used for infections' treatments and deemed a safe and beneficial approach, there is little information concerning the use and effectiveness of CP or enriched plasma fractions for hospitalized COVID-19 patients. To assess this, we analyzed quantitatively and qualitatively all published complete clinical trials using CP.

In this analysis, the mortality outcome presented the strongest evidence in favor of CP treatment. The respective sensitivity sub-analysis determined that only the study design originated some heterogeneity, indicating that the overall effect of CP in reducing mortality seems conclusive.

The results further indicated that CP accelerates the SARS-CoV-2 clearance from patients, most likely due to its neutralizing capacity, especially in critically ill patients. Effective viral clearance after CP administration has also been observed in severely ill patients with influenza and other viral infections (24–26). This clearance is likely dependent on the antibody titers present in the donor plasma. Our findings did not find evidence that higher titers were more effective in diminishing viral load, nor mortality. Besides neutralization, higher antibody titers could result in severe adverse reactions, such as antibody-dependent enhancement (ADE). ADE promotes the uptake of the virus-antibody complex by immune cells, contributing to the maintenance of inflammation and potentiating acute respiratory distress, which may be deadly (27, 28). Hence, estimating the optimal concentration of donor plasma antibody titers is critical to obtain the best clinical outcome possible, with the least probability of adverse reactions.

The results of the hospitalization period for CP treated patients, either severe or moderately ill, indicated a tendency for longer hospitalization, as compared to ST. This could be because CP patients with life-threatening/critical disease when able to survive, may have a lengthy recovery period as the clinical improvements occur at a slower pace.

We identified little evidence that CP reduced the need for non-invasive and invasive mechanical ventilation. Including more trials in this study could help clarify this tendency and obtain statistically supported results.

According to our results, only a minimal number of transfusion-associated adverse effects, common to routine blood transfusion, were observed after CP treatment. The severe adverse reactions that might occur after CP includes ADE, transfusion-related acute lung injury (TRALI) or transfusion-associated circulatory overload (TACO) (27, 28). Nevertheless, none of these types of effects was reported in our studies. Moreover, all patients that suffered from adverse reactions recovered without sequelae, after treatment. Thus, our results suggest that CP therapy can be deemed a safe therapy for COVID-19 since no major life-threatening events were reported. The safety of CP administration in COVID-19 patients is in concordance with other trials using CP as treatment for other infection types, such as Ebola and influenza (24, 29).

The use of concomitant medications (Supplementary Table 4) may have a synergistic effect with CP and further potentialized its therapeutic effect. Hence, comparing patients treated or not with CP may not suffice to exclude potential medication interactions in the evaluation of the different outcomes.

The safety and efficacy of CP treatment should soon be further clarified. According to the Clinicaltrials.gov 91 studies were initiated concerning the use of CP in SARS-CoV-2 infections, including at least 49 RCT (Supplementary Table 8). These ongoing trials will provide further data that may complement our findings. Also, they are assessing new primary and secondary outcomes variables, such as Sequential Organ Failure Assessment (SOFA) or changes in biomarkers during treatment. Therefore, soon the efficacy of CP as a front-line treatment for COVID-19 and propensity/severity of CP adverse events could be further corroborated.

Concerning the quality assessment of the clinical trials included in this meta-analysis, most RCT showed a low risk for bias, while CNRT presented serious or critical issues, which could bias the results. The most common issue is the lack of adjustment of their analysis for confounding variables. Yet, most CNRTs (14, 15, 17, 19, 21) mitigated bias by choosing the control group with matching characteristics, such as age, gender, and comorbidities to those of CP patients. Such bias does not immediately equate to poor study quality but rather determines the heterogeneity identified in some parameters. The quality of the non-randomized included trials is the aspect that could affect our meta-analysis results'. Nevertheless, the results were consistent with the overall findings, increased the assessments and the overall significance of the results.

## Conclusion

To date, the number of complete and available studies of clinical trials using CP as therapy for COVID-19 patients is scarce. Moreover, they differ, from the quantity of antibodies transfused to the types of co-medications administered and the sample size, which impacts their outcomes' results. Individually, most trials registered benefit in some outcomes but no overall advantageous effect of CP. Nevertheless, altogether, we verified strong evidence that CP therapy reduces mortality and efficient viral clearance. Although no definitive conclusions can be withdrawn from this meta-analysis, CP appears to be a safe therapy. To date, the Chai et al. (30) meta-analysis is the most detailed, despite only evaluating the data of randomized trials, which limited the appraisals performed. Yet, the few results they obtained showed a tendency favoring CP treatment. For a more robust assessment, we chose to include both types of studies in this study, though highlighting the constrictions on the interpretation of the results.

Currently, we observe the approval of several therapeutic drugs and vaccines for COVID-19 by the WHO and national regulators worldwide. Yet, it is not unlikely a re-emergence of infections with SARS-CoV-2 or its variants. Thus, CP could play a critical role in counterattacking new COVID-19 waves as a front-line treatment or as adjuvant therapy because it can be readily obtained, lessening the burden in the health care systems worldwide. This depends on the existence of enough scientific evidence supporting CP as a safe and effective treatment. In here lies the importance of systematic reviews and meta-analysis to impartial and critically evaluate the data from clinical trials.

## Data Availability Statement

The original contributions presented in the study are included in the article/Supplementary Material, further inquiries can be directed to the corresponding author/s.

## Author Contributions

DG, LS, and PV: study conception and design. DB and RL: data collection and analysis. DB and RC: tables preparation. DB: figures preparation. DB, RL, DG, LS, and PV: manuscript writing and revision. All authors have given approval to the final manuscript for submission.

## Conflict of Interest

The authors declare that the research was conducted in the absence of any commercial or financial relationships that could be construed as a potential conflict of interest.
