# Supplementary material for: Assessment of the Safety and Therapeutic Benefits of Convalescent Plasma in COVID-19 Treatment: A Systematic Review and Meta-Analysis
Source: Front Med (Lausanne). 2021 Apr 6;8:660688. doi: 10.3389/fmed.2021.660688 (PMC8055850; doi:10.3389/fmed.2021.660688)
Supplement: Supplementary file 1 [file Data_Sheet_1.docx]

Supplementary Material

# Contents

**Table 1**. Table indicating the studies not included for assessment and the justification.

**Table 2.** Table presenting the methodological characteristics of the studies included in the meta-analysis.

**Table 3**. Table presenting the baseline demographics of the studies included in the meta-analysis.

**Table 4.** Table presenting the interventions characteristics of the studies included in the meta-analysis.

**Table 5.** Table resuming major results of the studies included in the meta-analysis.

**Table 6**. Risk of bias assessment for the primary outcomes of the Randomized Controlled Trials (RCTs).

**Table 7.** Risk of bias assessment for the primary outcomes of the Controlled Non-Randomized Trials (CNRTs).

**Table 8**. Resume of the primary and secondary outcomes for each clinical trial included in this analysis.

**Figure 1.** Forest plot for length of hospitalization analysis assessing CP intervention and standard treatment.

**Figure 2.** Forest plot assessing the mortality rate exclusively in the studies included in the length of hospitalization outcome’s meta-analysis.

**eTable 1.** Table indicating the articles excluded for assessment and the justification.

| **Excluded articles** | **Title** | **Justification** |
| --- | --- | --- |
| Casadevall A. et al. 2020 | “A randomized Trial of Convalescent Plasma for COVID-19 – Potentially hopeful signals” | Editorial of a published randomized trial |
| Knudson CM. et al. 2020 | “COVID-19 convalescent plasma: phase 2” | Commentary |
| Zeng F. et al. 2020 | “Convalescent plasma for patients with COVID-19.” | Letter regarding Duan et al. 2020 trial |
| Im JH. Et al. 2020 | “Convalescent plasma therapy in coronavirus disease 2019: A case report and suggestions to overcome obstacles” | Case report  (non-controlled and non-randomized study) |
| Eckhardt CM. et al. 2020 | “Evaluating the efficacy and safety of human anti-SARS-CoV-2 convalescent plasma in severely ill adults with COVID-19: A structured summary of a study protocol for a randomized controlled trial” | Protocol |
| Joyner MJ. Et al. 2020 | “Early safety indicators of COVID-19 convalescent plasma in 5,000 patients” | Single-arm interventional study |
| Olivares-Gazca JC. Et al. 2020 | “Infusion of convalescent plasma is associated with clinical improvement in critically ill patients with covid-19: a pilot study” | Case report  (non-controlled and non-randomized study) |
| Erkurt MA. et al. 2020 | “Life-saving effect of convalescent plasma treatment in covid-19 disease: Clinical trial from eastern Anatolia” | Case series  (non-controlled and non-randomized study) |
| Perotti C. et al. 2020 | “Mortality reduction in 46 severe Covid-19 patients treated with hyperimmune plasma. A proof of concept single arm multicenter trial” | Single-arm interventional study |
| Shen C. et al. 2020 | “Treatment of 5 Critically Ill Patients With COVID-19 With Convalescent Plasma” | Case report  (non-controlled and non-randomized study) |
| Salazar E. et al. 2020 | “Treatment of Coronavirus Disease 2019 (COVID-19) Patients with Convalescent Plasma” | Case series  (non-controlled and non-randomized study) |
| Perotti C. et al. 2020 | “Plasma from donors recovered from the new Coronavirus 2019 as therapy for critical patients with COVID-19 (COVID-19 plasma study): a multicentre study protocol” | Protocol |
| Zhu M. et al. 2020 | “Use of convalescent plasma in COVID-19 patients in China” | Letter of CP use for COVID-19 patients in China |
| Ahn JY. Et al. 2020 | “Use of convalescent plasma therapy in two covid-19 patients with acute respiratory distress syndrome in Korea” | Case report  (non-controlled and non-randomized study) |
| Ye M. et al. 2020 | “Treatment with convalescent plasma for COVID-19 patients in Wuhan, China” | case report  (non-controlled and non-randomized study) |
| Zhang B. et al. 2020 | “Treatment with Convalescent Plasma for Critically Ill Patients with Severe Acute Respiratory Syndrome Coronavirus 2 Infection.” | case report  (non-controlled and non-randomized study) |
| Bobek I. et al. 2020 | “Successful administration of convalescent plasma in critically ill COVID-19 patients in Hungary: the first two cases” | case report  (non-controlled and non-randomized study) |
| Abdullah HM. et al. 2020 | “Severe refractory COVID-19 patients responding to convalescent plasma; A case series” | Not accessible |
| Jafari R. et al. 2020 | “Convalescent Plasma: An Old Trick for the Treatment of COVID-19” | Commentary |
| Zheng k. et al. 2020 | “A Scoping Review of Registered Clinical Trials of Convalescent Plasma for COVID-19 and a Framework for Accelerated Synthesis of Trial Evidence (FAST Evidence)” | Review |
| Islam A. et al. 2020 | “Convalescent plasma therapy in the treatment of COVID-19: Practical considerations: Correspondence” | Only mentions case reports and case series |
| Fragkou PC. et al. 2020  (1) | “Review of trials currently testing treatment and prevention of COVID-19” | No specific trials are mentioned |
| Rejeki MS. (PI) (1) | “Convalescent Plasma Therapy in Patients With COVID-19” | No results posted |
| Abdulamir AS. (PI) (1) | “Convalescent Plasma Therapy on Critically-ill Novel Coronavirus (COVID-19) Patients” | No results posted |
| Madariaga ML. (PI) (1) | “Convalescent Plasma Trial in COVID -19 Patients” | No results posted |
| Sevdi SSS. (PI) (1) | “Effectiveness of Convalescent Immune Plasma Therapy” | No results posted |
| Institute of Liver and Biliary Sciences, India (Sponsors and Collaborators) (1) | “Efficacy of Convalescent Plasma Therapy in Severely Sick COVID-19 Patients” | No results posted |
| Perotti C. (PI) (1) | “Hyperimmune Plasma for Critical Patients With COVID-19” | No results posted |

1. These are completed trials from the ClinicalTrials.gov database.

Table 2. Table presenting the methodological characteristics of the studies included in the meta-analysis.

| **Author** | **Published in**  **(and date)** | **Country** | **Type of study** | **Details about randomization** | **Details about allocation sequence concealment** | **Details about blinding** | **Methods used to prevent and control for confounding, selection biases, and information biases** | **Missing data** | **Bias tendency** |
| --- | --- | --- | --- | --- | --- | --- | --- | --- | --- |
| **Li L. et al. 2020** | JAMA  June 3 | China | RCT | Computer software | NR | open-label trial | To avoid assessment bias, the evaluation of clinical outcomes was performed by an investigator who was blind to the study group allocation | Missing data for secondary outcomes and adverse events were not imputed. Only observed values were used for data analysis and presentation | Yes, Dr Liu reports holding a pending patent on COVID-19 testing. Dr.Wu reports consulting for Verax Medical and Grifols, receiving royalties from UptoDate and AABB, and being a volunteer visiting professor and receiving travel support for giving medical education for the Chinese Institute of Blood Transfusion. |
| **Abolghasemi H. et al. 2020** | Transfusion and Apheresis Science  July 15 | Iran | CNRT | no randomization | NR | NR | NR | NR | none |
| **Zeng Q. et al. 2020** | The Journal of Infectious Diseases  April 29 | China | CNRT | no randomization | NR | NR | NR | NR | none |
| **Duan K. et al. 2020** | PNAS  April 28 | China | CNRT | no randomization | NR | NR | NR | NR | none |
| **Hegerova L. et al. 2020** | BLOOD  August 6 | USA | CNRT | no randomization | NR | NR | NR | NR | none |
| **Liu S. et al. 2020** | BLOOD  September 15 | USA | CNRT | no randomization | NR | "Chart review for clinical data unobtainable from the database was performed by a data team who were blinded to the cases to whom controls were matched" | "To confirm the independent effect of convalescent plasma transfusion on improvement in oxygenation and survival, we conducted a propensity score-matched analysis using The Mount Sinai Hospital’s COVID-19 confirmed patient pool from the same calendar period (march 24 to April 8)"  "A logistic regression was fit to predict the potential for plasma therapy based on time series data obtained at baseline upon admission, prior to transfusion, and the day of transfusion. Among the predictors, exact matching was enforced on the administration of hydroxychloroquine and azithromycin, intubation status and duration, length of hospital stay, and oxygen requirement on the day of transfusion. Other medications were administered too infrequently to enforce exact matching." "Selection bias is also a potential confounder, though propensity score matching was utilized to attempt to reduce selection bias by specifically selecting untreated controls with a similar propensity for treatment as those who were ultimately treated." "To the extent possible, EMR data were analysed objectively by computer algorithm without human involvement". | "Data that could not be obtained from discrete data fields was chart abstracted by individuals blinded to the case-control matches" | yes "F. Krammer has filed patent applications for the assay used to select plasma donors, and Mount Sinai has licensed its use to several companies" |
| **Gharbharan A. et al. 2020** | Medrxiv | Netherlands | RCT | web-based system | NR | open-label trial | NR | NR | NR |
| **Xia X. et al. 2020** | Blood  August 6 | China | CNRT | no randomization | NR | NR | NR | NR | NR |
| **Avendano-Sola et al. 2020** | Medrxiv  September 29 | Spain | RCT | web-based eCRF | it was concealed | not blinded | NR | NR | NR |
| **Agarwal et al. 2020** | BMJ  October 22 | India | RCT | RALOOC module in STAT v.14 by independent biostatistician (block randomization 1:1) | yes "…and called a member of the central trial coordinating team to receive the randomization sequence, enduring concealment of allocation." | open-label trial | NR | NR | yes, some investigators were working for the funding source |
| **Simonovich VA. Et al. 2020** | The New England Journal of Medicine  (November 24) | Argentina | RCT | REDCap® randomization program (in a 2:1 ratio), in variable size blocks of 3, 6, 9 and 12 participants and stratified by clinical site. | Participants and the entire clinical team, the data collectors, and the outcome adjudicators were unaware of the treatment assignments.  Except randomization investigators which oversaw the preparation of the infusion bags. | Double-blinding trial | NR | NR | NA |

Abbreviations: RCT-Randomized Controlled Trials | CNRT-Controlled Non-Randomized Trials | USA-United States of America | NR-Not reported | NA-Not assessable

Table 3. Table presenting the baseline demographics of the studies included in the meta-analysis.

| **Author** | **Study eligibility criteria** | **Disease severity at admission**  **(with definition if reported)** | **comorbidities (No. patients)** | **Major symptoms (No. patient)** | **Time of symptom onset until hospitalization, median (IQR)** | **Oxygen requirements at admission (types of supports and No. patients)** |
| --- | --- | --- | --- | --- | --- | --- |
| **Li L. et al. 2020** | **Inclusion criteria: (1)** signed informed consent; **(2)** aged at least 18 years; **(3)** COVID-19 diagnosis based on polymerase chain reaction (PCR) testing; **(4)** positive PCR result within 72 hours prior to randomization; **(5)** pneumonia confirmed by chest imaging; **(6)** clinical symptoms meeting the definitions of severe or life-threatening COVID-19; **(7)** acceptance of random group assignment; **(8)** hospital admission; **(9)** willingness to participate in all necessary research studies and be able to complete the study follow-up; and **(10)** no participation in other clinical trials, such as antiviral trials, during the study period. **Exclusion criteria:** **(1)**pregnancy or lactation; **(2)**immunoglobulin allergy; **(3)**IgA deficiency; **(4)**pre-existing comorbidity that could increase the risk of thrombosis; **(5)**life expectancy less than 24 hours; **(6)**disseminated intravascular coagulation; **(7)**severe septic shock; **(8)**PaO2/FIO2 of less than 100; **(9)**severe congestive heart failure; **(10)**detection of high titer of S protein–RBD-specific (receptor binding domain) IgG antibody (≥1:640); **(11)**other contraindications as determined by the patient’s physicians; and **(12)**participation in any antiviral clinical trials for COVID-19 within 30 days prior to enrolment. | severely or life-threatening ill  "Severe COVID-19 was defined as respiratory distress (≥30 breaths/min; in resting state, oxygen saturation of 93% or less on room air; or arterial partial pressure of oxygen (PaO2)/ fraction of inspired oxygen (FIO2) of 300 or less.  Life-threatening COVID-19 was defined as respiratory failure requiring mechanical ventilation; shock; or other organ failure (apart from lung) requiring intensive care unit (ICU) monitoring." | CP group Allergy n=6 Hypertension n=29 Cardiovascular disease n=14 Cerebrovascular disease n=11 Liver disease n=5 Diabetes n=9 Cancer n=3  Kidney disease n=2  Control group Allergy n=5 Hypertension n=27 Cardiovascular disease n=12 Cerebrovascular disease n=7 Liver disease n=5 Diabetes n=12 Cancer n=0 Kidney disease n=4 | NR | CP group 12 days  (5-20) [n = 49]^a^   Control group 10 days  (6-16) [n = 48]^a^ | Supplemental oxygen (not high flow or non-invasive) CP group n=15/51 Control group n=15/50   HFNCO and/or non-invasive mechanical ventilation CP group n=21/51 Control group n=23/50   ECMO and/or invasive mechanical ventilation CP group n=14/51 Control group n=11/50 |
| **Abolghasemi H. et al. 2020** | **Inclusion criteria: (1)**Age ≥ 18 years; **(2)**Confirmed COVID-19 infection through laboratory (RT-qPCR) and/or lung involvement confirmed with chest imaging (CT scan); **(3)**Presence of some or all of disease clinical symptoms such as shortness of breath (dyspnea), respiratory frequency ≥ 20/min, fever and cough; **(4)**Hospitalized with a blood oxygen saturation (SPO2) ≤93 % at rest on room air; **(5)**≤7 days since illness onset; **(6)**Willingness to participate in the trail and sign the consent form.  **Exclusion criteria:** **(1)**Intubated patients or patients on mechanical ventilation; **(2)**Severe liver or kidney disease; **(3)**Septic Shock; **(4)**Physician decision that convalescent plasma therapy is not in patients' best interest; **(5)**Patients with improving clinical conditions who meet hospital discharge criteria (defined as clinical recovery, i.e. return of body temperature, respiratory rate, oxygen saturation to normal and cough relief); **(6)**Known hypersensitivity to plasma. | CP group severely ill and Control group mostly mild disease | CP group Hypertension n=22 Diabetes n=27  Control group Hypertension n=19 Diabetes n=16 | NR, except Chest CT scan score on admission (mean):  CP group 13,81 (range 4-23)  Control group 13,36  (range 2-23) | NR | exclusion criteria of admission |
| **Zeng Q. et al. 2020** | **(1)** patients with laboratory confirmed COVID-19 diagnosed according to WHO;  **(2)** critically ill patients with COVID-19 that required intensive care unit admission | critically ill | CP group Hypertension n=1 Diabetes n=1 Cardiovascular diseases n=1 Chronic liver disease n=0 Respiratory system diseases n=0 Chronic kidney disease n=0  Control group Hypertension n=3 Diabetes n=5 Cardiovascular diseases n=0 Chronic liver disease n=2 Respiratory system diseases n=1 Chronic kidney disease n=1 | CP group -fever n=5 -cough n=5 -fatigue n=4 -shortness of breath n=4 -dyspenea n=3 -chest CT findings (bilateral pneumonia n=6; multiple mottling/ground-glass opacity n=5)  Control group -fever n=13 -cough n=14 -fatigue n=10 -shortness of breath n=12 -dyspenea n=8 -chest CT findings (bilateral pneumonia n=14; multiple mottling/ground-glass opacity n=14) | NR | HFNCO CP group n=6 Control group n=15  Mechanical ventilation CP group n=5 Control group n=13  ECMO CP group n=4 Control group n=12 |
| **Duan K. et al. 2020** | **Inclusion criteria** were one of the conditions 2 to 4 plus condition 1: **(1)** age ≥ 18 y; **(2)**respiratory distress, RR ≥30 beats/min; **(3)**oxygen saturation level less than 93% in resting state; **(4)**partial pressure of oxygen (PaO2)/oxygen concentration (FiO2) ≤ 300 mmHg (1 mmHg = 0.133 kPa).  **Exclusion criteria: (1)**previous allergic history to plasma or ingredients (sodium citrate); **(2)**cases with serious general conditions, such as severe organ dysfunction, who were not suitable for CP transfusion. | severely ill | CP group -hypertension n=3 -cardiovascular diseases n=1 -cerebrovascular diseases n=1  Control group NR | CP group -fever n=7 -cough n=8 -shortness of breath n=8 -sputum production 5 -chest pain n=2 -vomiting n=2 -nausea and vomiting/ sore throat/ diarrhea/ muscle ache/ athralgia/ headache n=1 each  Control group NR | CP group 6 day  (2.5-8.5)   Control group NR | Only reported for **CP group**  No oxygen support n=2  LFNCO n=2  HFNCO n=3  Mechanical ventilation n=3 |
| **Hegerova L. et al. 2020** | **(1)** severely and critically ill hospitalized COVID-19 patients; **(2)** diagnosed using quantitative reverse-transcriptase polymerase chain reaction assay for SARS-CoV-2 on nasopharyngeal swabs; | severely or life-threatening ill | CP group Hypertension n=12 Diabetes n=9 Obesity n=4  Control group NR | CP group -cough n=18 -shortness of breath n=18 -lymphopenia n=13 -abnormal radiograph (lung damage) n=16  Control group NR | NR | Mechanical ventilation to 1 /3 of patients |
| **Liu S. et al. 2020** | NR in the paper, only mention to FDA criteria  **FDA criteria:** **(1)**Laboratory confirmed COVID-19; **(2)**severe or life threatening COVID-19; **(3)** informed consent provided by the patient or healthcare proxy; **(4)** >= 18 years old | severely or life-threatening ill  (according to FDA recommendations)  **Severe disease** is defined as one or more of the following: shortness of breath (dyspnea); respiratory frequency ≥30/min; blood oxygen saturation ≤ 93%; partial pressure of arterial oxygen to fraction of inspired oxygen ratio <300; lung infiltrates >50% within 24 to 48 hours  **Life-threatening** disease is defined as one or more of the following: respiratory failure; septic shock; multiple organ dysfunction or failure | CP group -Asthma n=3 -Cancer n=2 -Diabetes mellitus n=8 -Obstructive sleep apnea n=2 -Chronic kidney disease n=1 -Chronic obstructive pulmonary disease n=1 -Obesity n=21 -Smoking n=7  Control matching 1:4 -Diabetes mellitus n=39 -Obstructive sleep apnea or chronic obstructive pulmonary disease n=5 -Obesity n=33 -Smoking n=30  Control matching 1:2 -Diabetes mellitus n=22 -Obstructive sleep apnea or chronic obstructive pulmonary disease n=3 -Obesity n=16 -Smoking n=18 | CP group -fever n=26 -shortness of breath n=26 -cough n=24 -diarrhea n=8 -sputum production n=3 -sore throat n=2  Control groups NR | 7 days | Only reported for **CP group**  Standard nasal cannula n=7  HFO, HFNC or BiPAP n=27  Mechanical ventilation n=4 |
| **Gharbharan A. et al. 2020** | **Inclusion criteria: (1)**at least 18 years old; **(2)**clinical COVID-19 disease proven by a positive SARS-CoV-2 RT-PCR test in the previous 96 hours to the study admission; **(3)**admitted to the hospital; **(4)**Written informed consent by patient or legal patient representative.  **Exclusion criteria: (1)**Patients with a documented IgA deficiency; **(2)**on mechanical ventilation for >96 hours; **(3)**Participation in another intervention trial on the treatment of COVID-19 that falls under the Dutch law human research (WMO) and in which individual patients are randomized to different treatment options | severely ill | CP group -Hypertension n=11 -Diabetes n=13 -Cardiac n=9 -Pulmonary n=12 -Cancer n=5 -Liver cirrhosis n=1 -Chronic kidney disease n=1  -Immunodeficiency n=5  Control group -Hypertension n=11 -Diabetes n=8 -Cardiac n=11 -Pulmonary n=11 -Cancer n=3 -Liver cirrhosis n=0 -Chronic kidney disease n=6 -Immunodeficiency n=6 | NR | 8 days | oxygen by mask or non-invasive ventilation or HFNCO CP group n=31 Control group n=34  Mechanical ventilation CP group n=5 Control group n=8 |
| **Xia X. et al 2020** | **Inclusion criteria: (1)** Laboratory confirmed cases; **(2)**patients with abnormalities in chest; **(3)**patients who did not improve significantly after standard treatment; **(4)**critically ill patients.  **Exclusion criteria:** Patients allergic to plasma contents; | Severe and Critically ill  (The severity degree of each patient was determined according to the clinical classification criterion in Diagnosis and Treatment Protocol for Novel Coronavirus Pneumonia released by the National Health Commission).  **Severe cases:** (1) Respiratory distress (≧30 breaths/ min); (2) Oxygen saturation≤93% at rest; (3) Arterial partial pressure of oxygen (PaO2)/ fraction of inspired oxygen (FiO2)≦300mmHg (lmmHg=0.133kPa). Cases with chest imaging that showed obvious lesion progression within 24-48 hours >50% shall be managed as severe cases.  **Critical cases:** (1) Respiratory failure and requiring mechanical ventilation; (2) Shock; (3) With other organ failure that requires ICU care. | CP group -Hypertension n=53 -Diabetes n=31 -Cardiovascular disease n=27 -Chronic obstructive pulmonary disease n=12 -Malignancy n=4 -Chronic liver diseases n=4 -Chronic renal disease n=4 -Immunodeficiency n=2 -Cerebrovascular disease n=12  Control group -Hypertension n=508 -Cardiovascular disease n=210 -Chronic obstructive pulmonary disease n=91 -Malignancy n=53 -Chronic liver diseases n=391 -Chronic renal disease n=33 -Immunodeficiency n=4 -Cerebrovascular disease n=75 | CP group -fever n=93 -cough n=83 -fatigue n=57 -shortness of breath n=28 -diarrhea n=4 -chest congestion n=24 -nausea or vomiting 2  Control group -fever n=984 -cough n=863 -fatigue n=564 -shortness of breath n=150 -diarrhea n=39 -chest congestion n=175 -nausea or vomiting n=13 | CP group 35 days  (18-40)    Control group 25 days (14-35) | No oxygen support CP group n=55/138 Control group n=675/1430  LFNCO CP group n=50/138 Control group n=469/1430  HFNCO and/or non-invasive mechanical ventilation CP group n=28/138 Control group n=224/1430  ECMO and/or invasive mechanical ventilation CP group n=2/138 Control group n=4/1430 |
| **Avendano-Sola et al. 2020** | **Inclusion criteria: (1)**hospitalized for laboratory-confirmed SARS-CoV-2 infection (RT-PCR) **(2)**either radiographic evidence of pulmonary infiltrates or clinical evidence plus SpO2 ≤94% on room air **(3)**within 12 days from the onset of symptoms (fever or cough).  **Exclusion criteria:** Patients were excluded if already on mechanical ventilation (invasive or non-invasive) or high flow oxygen devices | moderately ill | CP group -Hypertension n=20 -Diabetes n=12 -Cardiovascular disorder n=6 -Chronic lung diseases n=2 -Chronic kidney disease n=2 -Immunodeficiency n=2  Control group -Hypertension n=12 -Diabetes n=5 -Cardiovascular disorder n=9 -Chronic lung diseases n=8 -Chronic kidney disease n=2 -Immunodeficiency n=5 | NR | CP group 5.5 days (4-7)   Control group 5 days (2-7) | no oxygen  CP group n=10/38 Control group n=13/43  LFNO (supplemental O2 by nasal prongs or mask) CP group n=28/38 Control group n=30/43 |
| **Agarwal et al. 2020** | **Inclusion criteria: (1)**Patients at least 18 years with confirmed COVID-19 based on a positive SARS-CoV-2 RT-PCR test; **(2)**admitted to the participating hospitals; **(3)**moderately ill with either partial pressure of oxygen in arterial blood/fraction of inspired oxygen (PaO2/FiO2) ratio between 200-300 or respiratory rate >24/min with SpO2 < 93% on room air,17; **(4)**if matched donor CP was available at the point of enrolment.  **Exclusion criteria: (1)**Pregnant and lactating women; **(2)**patients with known hypersensitivity to blood products; **(3)**recipients of immunoglobulin in the last 30 days; **(4)**patients with conditions precluding infusion of blood products; **(5)** participants in any other clinical trials and critically ill patients with PaO2/ FiO2 <200 or shock (requiring vasopressors to maintain a mean arterial pressure (MAP)≥65 or MAP< 65) | moderately ill | CP group -Hypertension n=92 -Diabetes n=113 -Coronary artery disease n=15 -Chronic obstructive pulmonary disease n=8 -Chronic kidney disease n=8 -Cerebrovascular disease n=3 -Obesity n=16 -Tuberculosis n=9 -Smoking n=19 -Liver cirrhosis n=0 -Cancer n=1  Control group -Hypertension n=81 -Diabetes n=87 -Coronary artery disease n=17 -Chronic obstructive pulmonary disease n=7 -Chronic kidney disease n=9 -Cerebrovascular disease n=1 -Obesity n=17 -Tuberculosis n=10 -Smoking n=18 -Liver cirrhosis n=2 -Cancer n=0 | CP group -fever n=77 -cough n=149 -fatigue n=183/234 -shortness of breath n=215 -X-ray findings n=184  Control group -fever n=85 -cough n=167 -fatigue n=182 -shortness of breath n=208 -X-ray findings n=183 | 4 days for both groups  (3-7) | LFNO (supplemental O2 by nasal prongs or mask) CP group n=181 Control group n=180  HFNCO or non-invasive mechanical ventilation CP group n=54 Control group n=47  Mechanical ventilation and intubation CP group n=0 Control group n=1 |
| **Simonovich VA. Et al. 2020** | **Inclusion criteria: (1)**Hospitalized adults (at least 18 years of age); **(2)** positive RT-PCR assay of a respiratory tract sample that was positive for SARS-CoV-2; **(3)** radiologically confirmed pneumonia; **(4)** no previous directives rejecting advanced life support; **(5)** and at least one of the following severity criteria: oxygen saturation (SaO2)<93% at rest and breathing ambient air, a ratio of the partial pressure of oxygen (PaO2) to the fraction of inspired oxygen (FiO2)<300 mm Hg (PaO2:FiO2), or a Sequential Organ Failure Assessment (SOFA) or modified SOFA (mSOFA) score of two or more points above baseline status (scores range from 0 to 24, with higher scores indicating more severe disease).  **Exclusion criteria: (1)** pregnant or lactating patients; **(2)** patients of reproductive age who were not willing to use contraceptive measures for a period of 30 days after enrolment; **(3)** patients with a history of blood component allergies; **(4)** an infectious cause of pneumonia other than SARS-CoV-2; **(5)** a requirement for mechanical ventilation, multiorgan failure, or any other condition that would impede the provision of informed consent. | Severely ill | CP group  -Asthma n=9 -Hematologic cancer n=4  -Solid tumours n=23 -Diabetes n=40  -Hypertension n=111 -congestive heart failure n=8  -Thromboembolic disease n=5  -Chronic renal failure n=10 -Chronic obstructive pulmonary disease n=23 -Obesity n=104 -Smoking (current n=6 or previously n=101)  Control group  -Asthma n=5 -Hematologic cancer n=3  -Solid tumours n=11 -Diabetes n=21  -Hypertension n=48 -Congestive heart failure n=3  -Thromboembolic disease n=2  -Chronic renal failure n=4 -Chronic obstructive pulmonary disease n=2 -Obesity n=52 -Smoking (current n=6 or previously n=37) | NR | NR | LFNCO CP group n=146 Control group n=70  Venturi or nonrebreather mask CP group n=49 Control group n=16  HFNCO  CP group n=11 Control group n=7 |

Abbreviations: CP-Convalescent Plasma | IQR-Interquartile Range | CT-Computerized Tomography | LFNO-Low Flow Nasal Oxygen | LFNCO-Low Flow Nasal Canula Oxygen | HFNCO-High Flow Nasal Canula Oxygen | BiPAP-Bilevel Positive Pressure Airway | ECMO-Extracorporeal membrane oxygenation| NR-Not reported

Symbols: No.-number | ml-millilitre | n – number of patients | O2 – oxygen

^a^ In this parameter, Li S. et al. Had information missing for six patients, hence the indication in brackets the number of patients (n) in which the data is based.

Table 4. Table presenting the interventions characteristics of the studies included in the meta-analysis.

| **Author** | **Intervention** | **Comparison intervention** | **No. patients in each group** | **Dose^a^** | **Administration start** | **administration length/duration** | **Frequency** | **concomitant medications / co-interventions (No. patients treated with each medication)** |
| --- | --- | --- | --- | --- | --- | --- | --- | --- |
| **Li L. et al. 2020** | convalescent plasma administration | Standard treatment | CP group n=52  Control group n=51 | 4 to 13 mL/kg of recipient body weight  (median dose of 200ml  (IQR 200-300ml)) | median of 15 days since hospitalization | "10mL for the first 15 minutes, which was then increased to approximately 100 mL per hour with close monitoring" | One dose n=50  Two doses n=2 | CP group (in 46 patients) Antiviral n=41 Antibacterial n=38 Antifungal n=15 Steroids n=21 Human immunoglobulin n=13 Interferon n=12 Chinese herbal medicine n=26  Control group (in 49 patients) Antiviral n=44 Antibacterial n=39 Antifungal n=13 Steroids n=16 Human immunoglobulin n=11 Interferon n=7 Chinese herbal medicine n=30 |
| **Abolghasemi H. et al. 2020** | convalescent plasma administration | Standard treatment | CP group n=115  Control group n=74 | 500ml | less than 3 days after hospitalization | 4h | One dose, but if patients have no improvements in 24h, other unit could be administered | Antiviral (lopinavir/ritonavir, hydroxychloroquine); Anti-inflammatory agent  No. patients treated NR |
| **Zeng Q. et al. 2020** | convalescent plasma administration | Standard treatment | CP group n=6  Control group n=15 | median 300ml (IQR 200-600ml) | median 21.5 days during viral shedding (IQR 17.8-23) | NR | One dose n=3  Two doses n=3 | CP group Antiviral n=4 Antibiotics n=6 Intravenous immunoglobulins n=5 Glucocorticoid pulse n=4 Traditional Chinese medicine n=3 Continuous renal replacement therapy n=3  Control group Antiviral n=12 Antibiotics n=15 Intravenous immunoglobulins n=14 Glucocorticoid pulse n=12 Traditional Chinese medicine n=8 Continuous renal replacement therapy n=10 |
| **Duan K. et al. 2020** | convalescent plasma administration | Standard treatment | CP group n=10  control group n=10 | 200ml | median of 16.5d after symptoms onset (IQR 11-19.3) | 4h | One dose | CP group Antiviral (one drug or combined antiviral drugs) n=10 Antibacterial n=8 Antifungal n=2 Corticosteroids (intravenous methylprednisolone) n=6  Control group NR |
| **Hegerova L. et al. 2020** | convalescent plasma administration | Standard treatment | CP group n=20  control group n=20 | NR | median of 2 days after hospitalization  (IQR 1 -4.3) | NR | One dose | CP group Antiviral (hydroxycholoroquine) n=11 Antibiotics (azithromycin) n=12  Control group NR |
| **Liu S. et al. 2020** | convalescent plasma administration | Standard treatment | CP group n=39  Control 1:4 matching n=156  Control 1:2 matching n=74 | 250ml | median of 4 days since hospitalization | 1h to 2h | Two doses | CP group Antimicrobial agents (azithromycin n=31; broad spectrum n=29) Antivirals (hydroxychloroquine n=36; others n=1) Anti-inflammatory agents (corticosteroids 22; IL6 inhibitors n=3) Therapeutic anticoagulation n=26  Control group matching 1:4 Antimicrobial agents (azithromycin 133; broad spectrum 112) Antivirals (hydroxychloroquine 148; others 9) Anti-inflammatory agents (corticosteroids 90; IL6 inhibitors n=13) Therapeutic anticoagulation n=64  Control group matching 1:2 Antimicrobial agents (azithromycin n=63; broad spectrum n=57) Antivirals (hydroxychloroquine n=69; others n=4) Anti-inflammatory agents (corticosteroids n=38; IL6 inhibitors n=6) Therapeutic anticoagulation n=32 |
| **Gharbharan A. et al. 2020** | convalescent plasma administration | Standard treatment | CP group n=43  Control group n=43 | 300ml | median of 2 days since hospitalization (IQR 1-3) | NR | One dose n=41  Two doses n=2 | Antiviral (chloroquine, lopinavir/ritonavir) Anti-inflammatory (tocilizumab, anakinra) Antibiotics (azithromycin)  No. patients treated NR |
| **Xia X. et al 2020** | convalescent plasma administration | Standard treatment | CP group n=138  Control group n=1430 | 4-5ml/Kg  (200-400ml) | median of 45 days since disease onset | NR | One dose n=81 Two doses n=43 Three doses n=6 Four doses n=6 Five doses n=2 | NR |
| **Avendano-Sola et al. 2020** | convalescent plasma administration | Standard treatment | CP group n=38 but only 37 received   Control group n=43 | 250-300 ml | D1 after randomization (median of 3 days after hospitalization) | NR | One dose | CP group (in 38 patients) Antiviral (Hydroxycholoroquine n=34; Lopinavir-ritonavir n=15; Remdesivir n=1) Anti-inflammatory (tocilizumab n=10) Antibiotics (azithromycin n=24) Glucocorticoid therapy n=21 Low molecular weight heparin n=27  Control group (in 43 patients) Antiviral (Hydroxycholoroquine n=36; Lopinavir-ritonavir n=19; Remdesivir n=3) Antinflammatory (tocilizumab n=13) Antibiotics (azithromycin n=26) Glucocorticoid therapy n=25 Low molecular weight heparin n=33 |
| **Agarwal et al. 2020** | convalescent plasma administration | Standard treatment | CP group n=235   Control group n=229 | 200ml | D0 of randomization | NR | Two doses (24h apart) | CP group  Antiviral (Hydroxycholoroquine n=159; Lopinavir-ritonavir n=36; Remdesivir n=7) Anti-inflammatory (tocilizumab n=16) Antibiotics (azithromycin n=156; others n=204) Methylprednisolone n=123 Dexamethasone n=23 Hydrocortisone n=4 Intravenous immunoglobulin n=1 Heparin (UFL/LMWH) 178  Control group  Antiviral (Hydroxycholoroquine n=155; Lopinavir-ritonavir n=30; Remdesivir n=13) Anti-inflammatory (tocilizumab n=26) Antibiotics (azithromycin n=140; others n=196) Methylprednisolone n=114 Dexamethasone n=30 Hydrocortisone n=5 Intravenous immunoglobulin n=0 Heparin (UFL/LMWH) n=170 |
| **Simonovich VA. et al. 2020** | convalescent plasma administration | Placebo (normal saline solution) and Standard treatment | CP group n=228   Control group n=105 | median 500ml  (IQR 415-600ml)  “5-10ml/Kg with an inferior limit around 400ml for patients whose body weight was below 70 kg and a superior limit  of 600ml for those above 70 kg” | NR | 5-10 ml/kg/h | One dose | CP group  Antiviral (Hydroxycholoroquine n=1; Lopinavir-ritonavir n=7) Anti-parasitic (Ivermectin n=4) Anti-inflammatory (tocilizumab n=6)  Glucocorticoids n=209  Control group  Antiviral (Lopinavir-ritonavir n=3) Anti-parasitic (Ivermectin n=1) Anti-inflammatory (tocilizumab n=8)  Glucocorticoids n=93 |

Abbreviations: CP-Convalescent Plasma | IQR-Interquartile Range | NR-Not reported | indt-Indetermined

Symbols: No. – number | n – number of patients | ml – millilitre | Kg – kilogram

^a^ data presented in the indicated measures, except the cases indicated otherwise.

Table 5. Table resuming major results of the studies included in the meta-analysis.

| **Authors** | **Type** | **Severity and frequency**  **(No. patients)** | **Mortality assessment** | **Cause of death** | **Oxygen requirements during the study (types of supports and No. patients)** | **time until symptoms improvement** | **days for viral clearance (No. (%) negative results)** | **hospitalization period** | **place of hospital discharge** |
| --- | --- | --- | --- | --- | --- | --- | --- | --- | --- |
| **Li L. et al. 2020** | transfusion-associated adverse events | severe n=1 (severe transfusion-associated dyspnoea)  non-severe n=1 (non-severe allergic transfusion reaction (and probable non-severe febrile non-haemolytic transfusion reaction) ) | **D28**  CP group n=8/51 (severe 0; life-threatening 8/28)  Control group n=12/50: (severe patients 2/22; life-threatening patients 10/28) | NR | NR | CP group a median of 28 days;  Control group indt | **at 24h** CP group n=21/47 (44,7%) Control group n=6/40 (15%)  **at 48h** CP group n=32/47 (68,1%) Control group n=13/40 (32,5%)  **at 72h** CP group n=41/47 (87.2%) Control group n=15/40 (37.5%) | **median from randomization to discharge:** CP group 28 days Control group indt   **median from hospitalization to discharge:** CP group 41 days Control group 53 days   **At D28 had discharged:** CP group n=26/51 (51%) Control group n=18/50 (36%) | multicenter trial  (8 centers) |
| **Abolghasemi H. et al. 2020** | transient mild fever and chill | non-severe n=1 | **time point NR**  CP group n=17/115  Control group n=18/74 | NR | **Mechanical ventilation**  CP group n=8/115  Control group n=15/74 | NR | NR | **mean from hospitalization to discharge:** CP group 9,54days (range 2-24) Control group 12,88 days (range 2-32)   **mean from treatment to discharge:** CP group 6.25 days (range 0-20) Control group 12.88 days (range 2-32)   **During the study were discharged:** CP group n=98/115 (98,2%) Control group n=56/74 (78,7%) | multicenter trial  (6 centers) |
| **Zeng Q. et al. 2020** | none observed | ---------------------- | **time point NR**  CP group n=5/6  Control group n=14/15 | NR | NR | NR | **at 72h** CP group n=6/6 (100%)  Control group n=4/15 (26.7%) | NR | multicenter trial (2 centers) but mainly in the First Affiliated Hospital of Zhengzhou University |
| **Duan K. et al. 2020** | evanescent facial red spot | non-severe n=1 | **time point NR**  CP group n=0/10;  Control group n=3/10 | NR | **Before CP transfusion** No oxygen support n=1 LFNO n=2 HFNO n=3 Mechanical ventilation and HFNO n=2 Mechanical ventilation n=1  **After CP transfusion** No oxygen support n=3 LFNO and intermittent LFNO n=1 each HFNO n=4 Mechanical ventilation n=1 | began at 1 day after CP treatment. | CP group **at 24h** n=3/7 (42,9%) **at 48h** n=6/7 (85,7%) **at 120h** n=7/7 (100%)  (3 patients of the CP group were already negative before the CP transfusion)  Control group NR | NR | multicenter trial  (3 centers) |
| **Hegerova L. et al. 2020** | none observed | ---------------------- | **D7**  CP group n=2/20  Control group n=5/20  **D14**  CP group n=2/20  Control group n=6/20 | CP group the deceased patients had been intubated for >2weeks and choose comfort measures;  Control group NR | **Mechanical ventilation** D7 CP group n=6/20 Control group n=4/20  D14 CP group n=5/20 Control n=1/20 | NR | NR | **At D7 had discharged:** CP group n=5/20 (25%) Control group n=7/20 (35%)   **At D14 had discharged:** CP group n=9/20 (45%) Control group n=9/20 (45%) | multicenter trial (5 centers) |
| **Liu S. et al. 2020** | none observed | ---------------------- | **May 1**  CP group n=5/39  Control group matching 1:4 n=38/156  Control group matching 1:2 n=16/76 | "One patient died within 7d of transfusion, on day 3 after transfusion, of cardiac arrest in the setting of progressive hypoxemia due to COVID-19. The other four deaths during the study period, all due to COVID-19 complicated by multiorgan failure and shock, occurred on days 8, 9, 13 and 14 after transfusion."  Control group NR | NR    ("By day 14, clinical condition had worsened in 18.0% of the plasma patients and 24.3% in the control patients ") | NR, only mentioned the status at D14 after treatment | NR | **on May 1 had discharged:**  CP group n=28 (71,8%)  Control group n=104 (66,7%) | An academic medical center in New York City |
| **Gharbharan A. et al. 2020** | none observed | ---------------------- | **D15**  CP group n=6/43  Control group n=11/43 | NR | NR | NR, only mentioned the status at D15 after treatment | NR | Measures in HR (hazard ratio) | multicenter trial (14 centers) |
| **Xia X. et al. 2020** | minor allergic reactions (pruritus or erythema) | non-severe n=3 | **April 20**  CP group n=3/138  Control group n=59/1430 | NR | **D7** CP group Discharged or hospitalized but without oxygen support n=116/138 LFNO n=8/138 HFNO or non-invasive ventilation n=9/138 ECMO or invasive mechanical ventilation n=2/138   Control group NR | CP group median 10 days | CP group **at 72h** n=9/25 (36%) **at D7** n=17/25 (68%) **at D14** n=20/25 (80%)  Control group NR | **median from hospitalization to discharge:**  CP group 22 days  (IQR 16-30)  Control group 14 days (IQR 8-21) | Wuhan Huoshenshan Hospital, China |
| **Avendano-Sola et al. 2020** | infusion-related adverse events | grade 3 or 4 n=2 (severe) | **D15** CP group n=0/38  Control group n=4/43  **D29** CP group n=0/38  Control group n=4/43 | Control group 5 patients died of underlying diseases | **D15** CP group -No oxygen support (but hospitalized) n=4/38 -Oxygen support n=4/38  Control group -No oxygen support (but hospitalized) n=2/43 -Oxygen support n=2/43 -HFNO or non-invasive mechanical ventilation n=1/43  ECMO n=1/43   **D29** CP group -No oxygen support (but hospitalized) n=0/38 -Oxygen support n=4/38  Control group -No oxygen support (but hospitalized) n=0/43 -Oxygen support n=1/43 -HFNO or non-invasive mechanical ventilation n=2/43 -ECMO n=1/43 | CP group median 6.5 days (IQR 4-9)  Control group median 6 days (IQR 5-8) | This result was evaluated at D3, D5, D8, D11, D15 and D29. We only present D3 and D5 to match other time points  **at 72h** **swab** CP group n=9/33  Control group n=4/36  **blood** CP group n=28/35  Control group n=30/40   **at 120h** **swab** CP group n=14/32  Control group n=7/33  **blood** CP group n=32/34  Control group n=34/39 | **median from randomization to discharge:**  CP group 8.5 days  (IQR 6-13)  Control group 9 days  (IQR 6-11) | multicenter trial  (14 centers) |
| **Agarwal et al. 2020** | minor adverse effects | non-severe n=12 | **D28**  CP group n=34/235  Control group n=31/229 | CP group 3 patients POSSIBLY died of CP transfusion related complications, but it is unclear | **Non-invasive Mechanical ventilation** CP group n=31/227 Control group n=37/224 **Invasive Mechanical ventilation**  CP group n=19/227 Control group n=19/224 | NR | CP group **at 72h** n=79/184 **at D7** n=114/173   Control group **at 72h** n=67/183 **at D7** n=93/169 | **median from hospitalization to discharge:**  CP group 14 days  (IQR 10-19)  Control group 13 days (IQR 10-18) | multicenter  (39 centers) |
| **Simonovich VA. et al. 2020** | Infusion-related adverse events | Non-severe n=11  (minor allergic reactions and non-haemolytic febrile reaction) | **D30**  CP group n=25/228  Control group n=12/105 | NR | **Supplemental oxygen requirement** CP group n=5/228 Control group n=2/105 **Invasive ventilatory support** CP group n=19/228 Control group n=10/105 | Median 15 days for both groups  (CP group IQR 9-ND;  Control group IQR 7-ND) | NR | **median from treatment to discharge:**  CP group 13 days (IQR 8-30)  Control group 12 days  (IQR 7-30) | multicenter  (12 centers) |

Abbreviations: CP-Convalescent Plasma | IQR-Interquartile Range | LFNO-Low Flow Nasal Oxygen | LFNCO-Low Flow Nasal Canula Oxygen | HFNCO-High Flow Nasal Canula Oxygen | BiPAP-Bilevel Positive Pressure Airway | ECMO-Extracorporeal membrane oxygenation| NR-Not reported | indt-Indetermined | ND-not determined

Symbols: No. – number | n – number of patients

**Table 6.** Risk of bias assessment for the primary outcomes of the Randomized Controlled Trials (RCTs). The risk of bias was evaluated using the Cochrane Library RoB2.0 tool, specific for RCTs. Legend bellow the table, including the judgment code and the risk of bias domains denomination. The D1 and D2 domains refer to study-level bias, whereas the D3-D5 domains concern the outcome bias.

|  | **Studies** | **D1** | **D2** | **D3** | **D4** | **D5** | **Overall bias** |
| --- | --- | --- | --- | --- | --- | --- | --- |
| **Mortality** | Agarwal et al. 2020 | 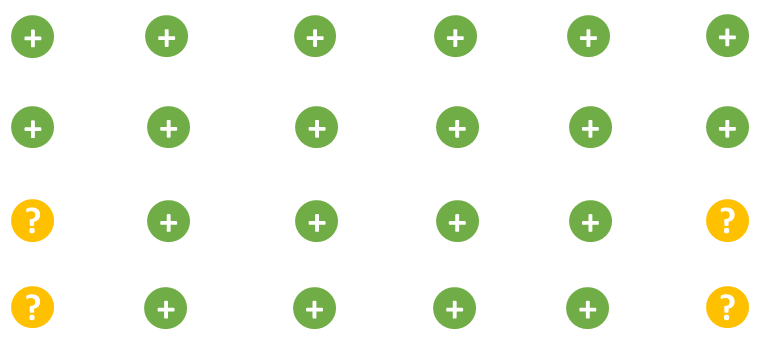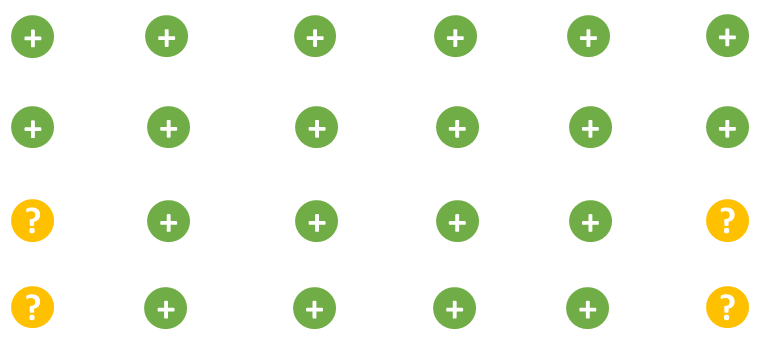 |  |  |  |  |  |
|  | Gharbharan et al. 2020 |  |  |  |  |  |  |
|  | Simonovich et al. 2020 |  |  |  |  |  |  |
| **Clinical status** | Agarwal et al. 2020 | 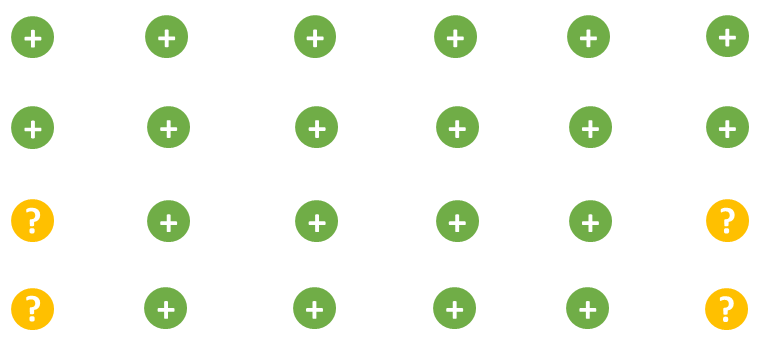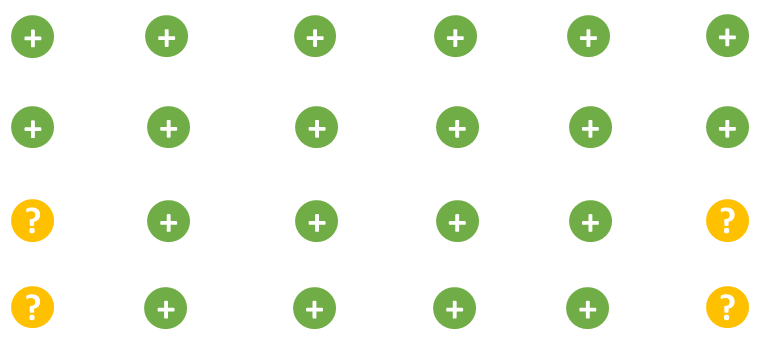 |  |  |  |  |  |
|  | Avendano-Sola et al. 2020 |  |  |  |  |  |  |
|  | Li et al. 2020 |  |  |  |  |  |  |
|  | Simonovich et al. 2020 |  |  |  |  |  |  |


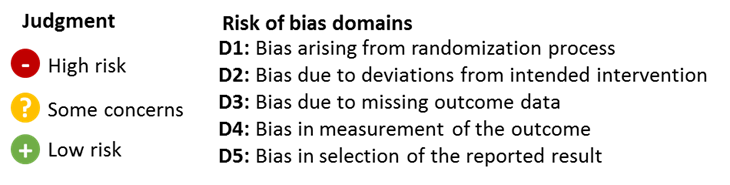


**Table 7.** Risk of bias assessment for the primary outcomes of the Controlled Non-Randomized Trials (CNRTs). The risk of bias was evaluated using the Cochrane Library ROBINS-I tool, specific for CNRTs. Legend bellow the table, including the judgment code and the risk of bias domains denomination. The D1-D4 domains refer to study-level bias, whereas the D5-D7 domains concern the outcome bias.

|  | **Studies** | **D1** | **D2** | **D3** | **D4** | **D5** | **D6** | **D7** | **Overall bias** |
| --- | --- | --- | --- | --- | --- | --- | --- | --- | --- |
| **Mortality** | Abolghasemi et al. 2020 | 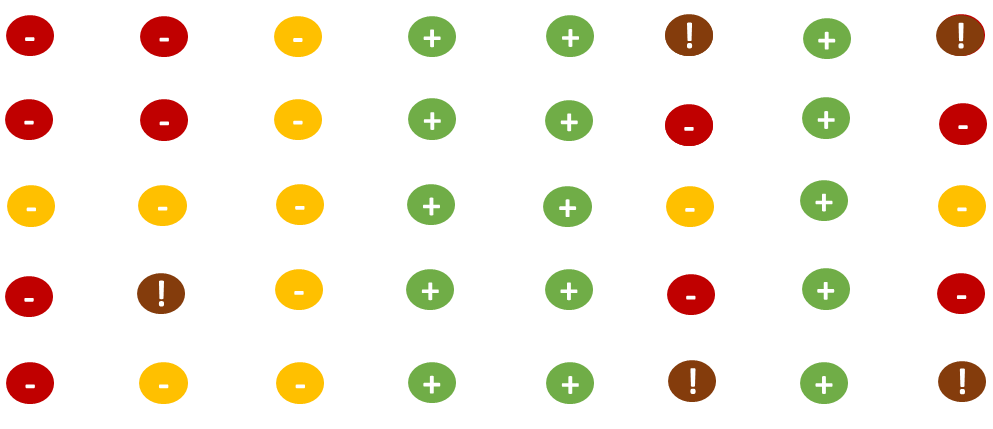 |  |  |  |  |  |  |  |
|  | Hegerova et al. 2020 |  |  |  |  |  |  |  |  |
|  | Liu et al. 2020 |  |  |  |  |  |  |  |  |
|  | Xia et al. 2020 |  |  |  |  |  |  |  |  |
|  | Zeng et al. 2020 |  |  |  |  |  |  |  |  |
| **Safety** | Duan et al. 2020 |  |  |  | 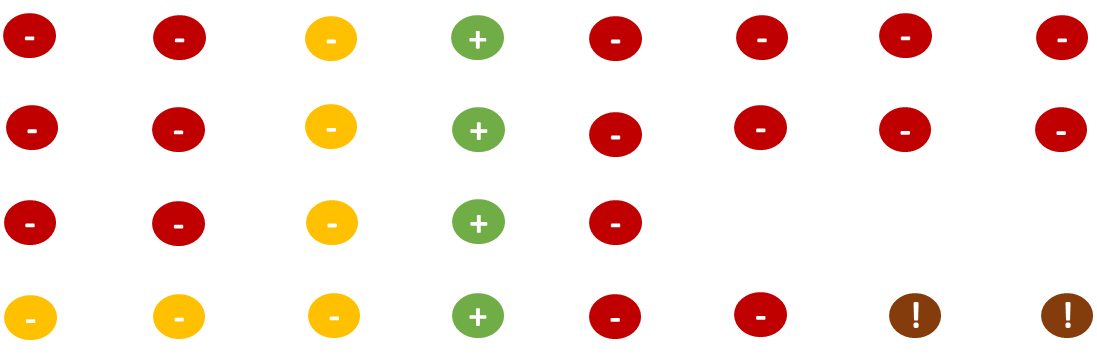 |  |  |  |  |
| **Length of hospitalization** | Abolghasemi et al. 2020 | 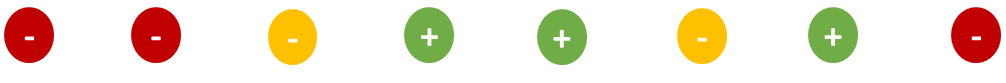 |  |  |  |  |  |  |  |


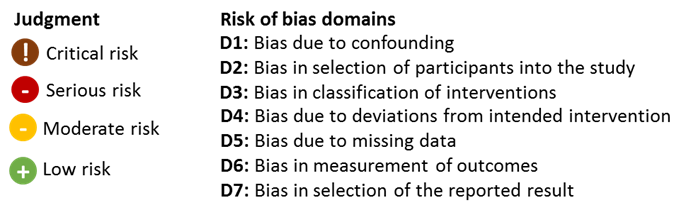


Table 8. Resume of the primary and secondary outcomes for each clinical trial included in this analysis. Data obtained from ClinicalTrials.gov.

| **NCT Number** | **Title** | **Primary Outcome Measures** | **Secondary Outcome Measures** |
| --- | --- | --- | --- |
| **NCT04433910** | A Clinical Trial of Convalescent Plasma Compared to Best Supportive Care for Treatment of Patients With Severe COVID-19 | Recovery | (1) Clinical improvement; (2) Adverse events; (3) Mortality; (4) Hospitalization;  (5) Length of stay in ICU; (6) Ventilation support/ECMO; (7) SARS-CoV-2 PCR negatives; (8) Comorbidities; (9) Coagulation markers; (10) Inflammation;  (11) Percentage of patients willing to donate plasma; (12) Amount of Plasma Units collected; (13) Titer of anti-SARS-CoV-2; (14) Impact of donor characteristics on anti-SARS-CoV-2 humoral response; (15) Correlation of anti-SARS-CoV-2 titer in transfused plasma units and primary and key secondary outcomes.; (16) Effect of timing of plasma transfusions. |
| **NCT04374487** | A Phase II, Open Label, Randomized Controlled Trial to Assess the Safety and Efficacy of Convalescent Plasma to Limit COVID-19 Associated Complications | (1) Severe ARDS;  (2) Mortality | (1) Symptom resolution; (2) Hospitalization; (3) Change in SOFA; (4) Respiratory support required; (5) Radiological improvement; (6) Adverse events (AE) associated with transfusion; (7) Change in RNA levels of SARS-CoV-2 from RT-PCR; (8) Bio-markers; (9) Need of Vasopressor use. |
| **NCT04418518** | A Trial of CONvalescent Plasma for Hospitalized Adults With Acute COVID-19 Respiratory Illness (CONCOR-1) | (1) In-hospital mortality; (2) Mechanical ventilation | Mechanical ventilation. |
| **NCT04383535** | Convalescent Plasma and Placebo for the Treatment of COVID-19 Severe Pneumonia | Clinical status at 30th day of follow-up | (1) Clinical status; (2) Hospitalization; (3) ICU length of stay; (4) Functional recovery; (5) Adverse events/serious adverse events; (6) Negative of SARS-CoV-2 load; (7) D-Dimer plasma concentration; (8) Ferritin plasma concentration; (9) Neutralizing antibodies; (10) Adverse reactions. |
| **NCT04442191** | Convalescent Plasma as a Possible Treatment for COVID-19 | Oxygen supplementation | (1) Mortality; (2) ICU admission; (3) Intubation; (4) Hospitalization; (5) Type/Length of respiratory support; (6) C-reactive Protein (CRP); (7) Lymphocyte red; (8) Lactate dehydrogenase; (9) Ferritin; (10) D-Dimer; (11) White Blood Cell count. |
| **NCT04342182** | Convalescent Plasma as Therapy for Covid-19 Severe SARS-CoV-2 Disease (CONCOVID Study) | Mortality | (1) Hospitalization; (2) Mortality; (3) ICU Length of stay; (4) SARS-CoV2 shedding from airways; (5) Impact of CTL and NK cell immunity on the likelihood of being protected from immune serum transfer; (6) Safety of CP therapy; (7) Change of the 8-point WHO COVID19 disease severity scale; (8) Impact of plasma therapy on risk of long-term structural lung damage and lung function. |
| **NCT04376034** | Convalescent Plasma Collection and Treatment in Pediatrics and Adults | Survival | (1) Incidence of treatment-emergent Adverse Events [Safety and Tolerability];  (2) Morbidity; (3) Length of Stay in hospital (4) Length of Stay on Advance Respiratory Support. |
| **NCT04395170** | Convalescent Plasma Compared to Anti-COVID-19 Human Immunoglobulin and Standard Treatment (TE) in Hospitalized Patients | (1) Admission to ICU;  (2) Mechanical ventilation | (1) Hospitalization; (2) Neutralizing antibody (IgG) titers against COVID-19;  (3) Adverse events; (4) Mortality. |
| **NCT04358783** | Convalescent Plasma Compared to the Best Available Therapy for the Treatment of SARS-CoV-2 Pneumonia | (1) Mortality;  (2) Time for SARS-CoV-2 RT-PCR negatives;  (3) Anti-SARS-CoV-2 antibody titers;  (4) Serum antibodies | (1) SARS-CoV-2 RT-PCR negatives; (2) Anti-SARS-CoV-2 antibody titers;  (3) Serum antibodies. |
| **NCT04390503** | Convalescent Plasma for COVID-19 Close Contacts | Severe Disease | (1) Anti-SARS-CoV-2 titers; (2) SARS-CoV-2 PCR positives; (3) Duration of SARS-CoV-2 PCR positives; (4) Levels of SARS-CoV-2 RNA. |
| **NCT04348656** | CONvalescent Plasma for Hospitalized Adults With COVID-19 Respiratory Illness (CONCOR-1) | (1) Intubation;  (2) In-hospital mortality | (1) Ventilation; (2) Mortality; (3) Adverse events. |
| **NCT04385199** | Convalescent Plasma for Patients With COVID-19 | Improvement in respiratory disease | Mortality. |
| **NCT04332835** | Convalescent Plasma for Patients With COVID-19: A Randomized, Open Label, Parallel, Controlled Clinical Study | (1) Viral Load;  (2) Immunoglobulin M COVID-19 Titers;  (3) Immunoglobulin G COVID-19 Titers | (1) ICU Admission; (2) ICU length of stay; (3) Hospitalization; (4) Mechanical ventilation; (5) Clinical status assessed according to the World Health Organization guideline; (6) Mortality. |
| **NCT04391101** | Convalescent Plasma for the Treatment of Severe SARS-CoV-2 (COVID-19) | (1) In-hospital mortality; (2) Length of hospital stay | (1) Mortality; (2) Hospitalization; (3) ICU length of stay; (4) Ventilatory support;  (5) Viral load; (6) Immunological response; (5) Negative COVID-19 load; (6) Donor Interferon Gamma profile characterization; (7) Donor Granulocyte Macrophage Colony Stimulating Factor characterization; (8) Donor Tumor Necrosis Factor Alfa characterization; (9) Donor Interleukin -1 beta characterization; (10) Donor Interleukin-2 characterization; (11) Donor Interleukin-4 characterization. |
| **NCT04405310** | Convalescent Plasma of Covid-19 to Treat SARS-COV-2 a Randomized Doble Blind 2 Center Trial | Mortality | (1) Length of stay ICU; (2) Mechanical Ventilation; (3) Oxygen support; (4) Viral Load by RT-PCR; (5) Inflammatory biomarkers; (6) SOFA score. |
| **NCT04403477** | Convalescent Plasma Therapy in Severe COVID-19 Infection | (1) In-hospital mortality; (2) Time to death | (1) Fever; (2) Respiratory distress; (3) Saturation of oxygen; (4) Blood pressure;  (5) Oxygen requirement; (6) C-reactive Protein; (7) Ferritin; (8) SGPT; (9) SGOT; (10) ICU length of stay; (11) Ventilator support; (12) Hospitalization; (13) Transfusion reaction. |
| **NCT04441424** | Convalescent Plasma Therapy on Critically-ill Novel Coronavirus (COVID-19) Patients | Mortality | Hospitalization. |
| **NCT04345523** | Convalescent Plasma Therapy vs. SOC for the Treatment of COVID19 in Hospitalized Patients | Category Changes in Ordinal Scale | (1) Time to category 5, 6 or 7 of the ordinal scale; (2) Mortality; (3) Oxygenation;  (4) Ventilator free days; (5) Treatment-Emergent Adverse Events; (6) Antibodies levels; (7) Viral load. |
| **NCT04364737** | Convalescent Plasma to Limit COVID-19 Complications in Hospitalized Patients | Score on WHO 11-point ordinal scale for clinical improvement | (1) Score on the WHO 11-point ordinal scale for clinical improvement at 28 days; (2) Anti-SARS-CoV-2 antibody titers (IgM, IgG, IgA); (3) SARS-CoV-2 PCR positive results; (4) Rate of mortality; (5) patients requiring ICU admission; (6) Lymphocyte counts; (7) Neutrophil counts; (8) D-dimer; (9) Fibrinogen; (10) T cell subsets; (11) B cell subsets. |
| **NCT04373460** | Convalescent Plasma to Limit SARS-CoV-2 Associated Complications in Hospitalized patients | (1) Hospitalization;  (2) Mortality;  (3) Serious adverse events;  (4) Treatment-related grade 3 or higher adverse events | (1) Serum SARS-CoV-2 antibody titers; (2) SARS-CoV-2 Polymerase Chain Reaction (PCR) negative results. |
| **NCT04323800** | Convalescent Plasma to Stem Coronavirus (CSSC-001) | Disease severity | (1) Anti-SARS-CoV-2 titers; (2) SARS-CoV-2 PCR positive results; (3) Duration of SARS-CoV-2 PCR positivity; (4) Peak quantity levels of SARS-CoV-2 RNA;  (5) Disease severity. |
| **NCT04356534** | Convalescent Plasma Trial in COVID -19 Patients | Requirement for invasive ventilation | (1) Viral clearance; (2) Radiological change; (3) White cell count; (4) C-reactive protein; (5) Lactate dehydrogenase; (6) Procalcitonin; (7) D-Dimer; (8) Ferritin;  (9) Troponin-T; (10) Brain naturetic peptide; (11) Mortality. |
| **NCT04381858** | Convalescent Plasma vs Human Immunoglobulin to Treat COVID-19 Pneumonia | (1) Hospitalization time; (2) Oxygenation index evolution;  (3) Severe Acute respiratory distress syndrome (ARDS);  (4) Mortality;  (5) Invasive mechanical ventilation | (1) Viral PCR negative; (2) Intubation; (3) Ventilation; (4) In-hospital mortality;  (5) Mortality; (6) Length of stay in ICU; (7) Hospitalization; (8) ECMO; (9) Renal replacement therapy; (10) Myocarditis; (11) Adverse events and serious adverse events; (12) CCP transfusion-associated adverse events. |
| **NCT04355767** | Convalescent Plasma vs. Placebo in Emergency Room Patients With COVID-19 | Disease progression; | Symptom severity over time. |
| **NCT04344535** | Convalescent Plasma vs. Standard Plasma for COVID-19 | Ventilator free days | Mortality. |
| **NCT04397757** | COVID-19 Convalescent Plasma for the Treatment of Hospitalized Patients With Pneumonia Caused by SARS-CoV-2. | (1) Serious adverse events;  (2) Severity score | (1) Clinical status assessment, using 8-point ordinal scale; (2) Clinical status assessment using the National Early Warning Score (NEWS); (3) Oxygenation;  (4) Ventilator/ECMO; (5) Hospitalization; (6) Mortality; (7) SAEs ;(8) Adverse events; (9) Changes in WBC; (10) Changes in Hemoglobin measurement through Day 29 of convalescent plasma administration by comparing treatment vs control arms;  (11) Changes in platelets; (12) Changes in creatinine; (13) Changes in glucose;  (14) Changes in bilirubin; (15) Changes in ALT measurement laboratory adverse events; (16) Changes in AST measurement; (17) Changes in PT measurement laboratory adverse events. |
| **NCT04384588** | COVID19-Convalescent Plasma for Treating Patients With Active Symptomatic COVID 19 Infection (FALP-COVID) | (1) In-hospital mortality; (2) Safety of the use of CP | (1) Mortality; (2) In-hospital Mortality; (3) Hospitalization; (4) ICU length of stay;  (5) Mechanical ventilation; (6) Viral load measuring and negative COVID-19 results; (7) Immunological response (COVID19-Immunoglobulin M and Immunoglobulin G, neutralizing antibodies); (8) Donor profile characterization; (9) Receptor profile characterization. |
| **NCT04374526** | Early transfusion of Convalescent Plasma in Elderly COVID-19 Patients. to Prevent Disease Progression. | COVID-19 progression | NR |
| **NCT04425837** | Effectiveness and Safety of Convalescent Plasma in Patients With High-risk COVID-19 | (1) Mortality;  (2) Adverse events | (1) ICU admission; (2) Mechanical ventilation; (3) ICU length; (4) D-Dimer; (5) LDH; (6) Troponin level; (7) Ferritin; (8) Procalcitonin; (9) CRP; (10) Lymphocyte count; (11) PaO2/Fio2; (12) SOFA score; (13) ECMO; (14) Lung infiltration. |
| **NCT04380935** | Effectiveness and Safety of Convalescent Plasma Therapy on COVID-19 Patients With Acute Respiratory Distress Syndrome | Mortality; | (1) Length of stay in ICU; (2) Mechanical ventilation; (3) Body temperature; (4) SOFA Score; (5) PAO2/FIO2 ratio; (6) C-Reactive Protein; (7) D-Dimer; (8) Procalcitonin; (9) Interleukin 6; (10) Allergic/ anaphylaxis transfusion reaction; (11) Hemolytic transfusion reaction; (12) Transfusion Related Acute Lung Injury (TRALI); (13) Transfusion associated Circulatory Overload (TACO). |
| **NCT04421404** | Effects of COVID-19 Convalescent Plasma (CCP) on Coronavirus-associated Complications in Hospitalized Patients | Severe Hypoxemic Respiratory Failure Endpoint | 8-Point Ordinal Scale Endpoint. |
| **NCT04375098** | Efficacy and Safety of Early COVID-19 Convalescent Plasma in Patients Admitted for COVID-19 Infection | (1) Mechanical ventilation;  (2) Hospitalization time | (1) Fever; (2) Mechanical ventilation; (3) ICU length of stay; (4) In-hospital mortality; (5) Mortality; (6) Readmission; (7) Viral clearance. |
| **NCT04425915** | Efficacy of Convalescent Plasma Therapy in Patients With COVID-19 | Clinical improvement | (1) Oxygenation; (2) Hospitalization; (3) Mechanical ventilation; (4) ICU length of stay; (5) Adverse effects; (6) Antibodies against SARS-CoV-2 in serum;  (7) Cytokines; (8) Acute phase reactants; (9) Correlation of the titers in COVID-19 convalescent plasma donors with duration of illness, severity of symptoms, duration of hospital stay, drugs used in therapy, duration between recovery, and donation. |
| **NCT04346446** | Efficacy of Convalescent Plasma Therapy in Severely Sick COVID-19 Patients | Mechanical ventilation | (1) Mortality; (2) IPa02/Fi02 ratio; (3) SOFA score; (4) Hospitalization; (5) ICU stay; (6) Requirements of Vasopressor; (7) Dialysis. |
| **NCT04372979** | Efficacy of Convalescent Plasma Therapy in the Early Care of COVID-19 Patients. | Survival time without needs of a ventilator | (1) Morbidity; (2) Mortality; (3) Hospitalization; (4) Viral pharyngeal specimen clearance; (5) Viral blood specimen clearance; (6) Hemostasis disorders;  (7) Neutralizing antibodies; (8) Transfusion endotheliopathy effect; (9) Transfusion biological Inflammation effect; (10) Transfusion hemovigilance; (11) Decrease in the consumption of antibiotics. |
| **NCT04345991** | Efficacy of Convalescent Plasma to Treat COVID-19 Patients, a Nested Trial in the CORIMUNO-19 Cohort | (1) Survival without ventilator;  (2) WHO progression scale ≥6 | (1) Severe adverse events; (2) WHO progression scale; (3) Survival;  (4) Hospitalization; (5) Oxygenation; (5) Survival without use of ventilator;  (6) Survival without use of immunomodulatory drugs. |
| **NCT04377568** | Efficacy of Human Coronavirus-immune Convalescent Plasma for the Treatment of COVID-19 Disease in Hospitalized Children | Clinical recovery | (1) Combined mortality/intubation; (2) Respiratory status; (3) Mortality; (4) Care and Critical Care; (5) Transfusion-associated adverse events; (6) Safety of the intervention. |
| **NCT04438057** | Evaluating the Efficacy of Convalescent Plasma in Symptomatic Outpatients Infected With COVID-19 | Time to resolution of Symptoms | (1) Inflammatory Markers; (2) Hospitalization. |
| **NCT04361253** | Evaluation of SARS-CoV-2 (COVID-19) Antibody-containing Plasma therapy | Modified WHO Ordinal Scale (MOS) score | (1) Need for Intubation; (2) Time to intubation; (3) Ventilation; (4) In-hospital mortality; (5) Mortality; (6) Length of stay in ICU; (7) Hospitalization; (8) ECMO;  (9) Renal replacement therapy; (10) Myocarditis; (11) Adverse events and serious adverse events; (12) CCP transfusion-associated adverse events. |
| **NCT04392414** | Hyperimmune Convalescent Plasma in Moderate and Severe COVID-19 Disease | Body temperature | (1) Mechanical ventilation; (2) Oxygenation; (3) ICU length of stay; (4) Changes of the titer of the SARS-CoV-2 antibodies in the blood plasma; (5) Cytokine profile;  (6) Cytokine storm development and the need of cytokine storm inhibitors; (7) CRP; (8) Mortality rate. |
| **NCT04385043** | Hyperimmune Plasma in Patients With COVID-19 Severe Infection | Mortality | (1) Lymphocyte count; (2) PCR levels; (3) AB levels and clinical improvement;  (4) Inflammatory cytokines. |
| **NCT04385186** | Inactivated Convalescent Plasma as a Therapeutic Alternative in Patients CoViD-19 | Mortality | Viral PCR Negative results. |
| **NCT04362176** | Passive Immunity Trial of Nashville II for COVID-19 | COVID Ordinal Outcomes Scale; | (1) Mortality; (2) COVID Ordinal Outcomes Scale; (3) Mortality/ECMO;  (4) Oxygenation; (5) Vasopressor; (6) Ventilator; (7) ICU; (8) Hospitalization. |
| **NCT04359810** | Plasma Therapy of COVID-19 in Critically Ill Patients | Time to Improvement; | (1) SARS-CoV-2 PCR Positive results; (2) Oxygenation; (3) Hospitalization; (4) In-hospital Mortality. |
| **NCT04347681** | Potential Efficacy of Convalescent Plasma to Treat Severe COVID-19 and Patients at High Risk of Developing Severe COVID-19 | (1) ICU length of stay;  (2) Safety of CP;  (3) Serious adverse reactions | (1) Mechanical ventilation; (2) Mortality ;(3) Clinical recovery. |
| **NCT04388410** | Safety and Efficacy of Convalescent Plasma Transfusion for Patients With COVID-19 | (1) Severity;  (2) Mortality;  (3) Adverse events | (1) Clinical improvement; (2) Antibodies against SARS-CoV-2; (3) Disease progression; (4) Mechanical ventilation; (5) Fever; (6) Adverse events. |
| **NCT04428021** | Standard or Convalescent Plasma in Patients With Recent Onset of COVID-19 Respiratory Failure | Survival | (1) Ventilation; (2) Mortality; (3) Complications; (4) ICU length of stay;  (5) Immunoglobulin G; (6) Clearance of viral load; (7) SOFA score; (8) Any variation from Standard Therapy Protocol. |
| **NCT04333251** | Study Testing Convalescent Plasma vs Best Supportive Care | (1) Oxygenation;  (2) Ventilation support | NR |
| **NCT04393727** | Transfusion of Convalescent Plasma for the Early Treatment of Patients With COVID-19 | Mechanical ventilation | (1) Mortality; (2) Invasive mechanical ventilation; (3) Virologic cure; (4) Hospitalization; (5) Adverse events. |
| **NCT04415086** | Treatment of Patients With COVID-19 With Convalescent Plasma | Time elapsed until clinical improvement or hospital discharge | (1) Acute adverse events; (2) Clinical Status; (3) Duration of clinical events;  (4) SARS-CoV-2 in nasopharyngeal swab; (5) IgG, IgM and IgA titers for SARS-CoV-2; (6) Neutralizing antibodies. |
| **NCT04432103** | Treatment of Severe and Critical COVID-19 Pneumonia With Convalescent Plasma | (1) Critical pneumonia;  (2) Mortality | Mechanical ventilation. |
| **NCT04408040** | Use of Convalescent Plasma for COVID-19 | (1) Mortality;  (2) Survival;  (3) Disease progression to severe or critical;  (4) Rate of infection among healthy health care providers | NR |
| **NCT04438694** | Assessment of the Effect of Convalescent Plasma Therapy in Patients With Life-threatening COVID19 Infection (CP IN COVID19) | (1) Hospitalization;  (2) Recovery status | NR |

Abbreviations: ALT-alanine aminotransferase | AST-aspartate aminotransferase | AB-antibody | CRP-C-reactive protein | CP-Convalescent Plasma | CCP-COVID-19 convalescent plasma | ECMO-Extracorporeal membrane oxygenation| ICU-intensive care unit | SOFA-Sequential Organ Failure Assessment | NR-Not reported | PaO2/Fio2- partial pressure of oxygen/oxygen concentration | SAE-serious adverse events | SGPT-serum glutamic pyruvic transaminase | SGOT- glutamic-oxaloacetic transaminase WVC-white blood cell.

**
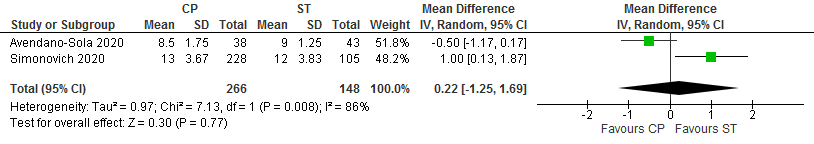
Figure 1** **– Forest plot for length of hospitalization analysis assessing CP intervention and standard treatment**. Length of hospitalization since beginning of treatment until discharge. The result showed no statistical difference between intervention groups, although high levels of heterogeneity are presented. No sub-analysis could be performed due to paucity of trials in evaluation.

The results are presented in mean and standard deviation (SD). Different sizes of data markers correspond to the relative weight assigned in the pooled analysis. Diamond marker indicates the overall result.

**(A)**

**(B)**


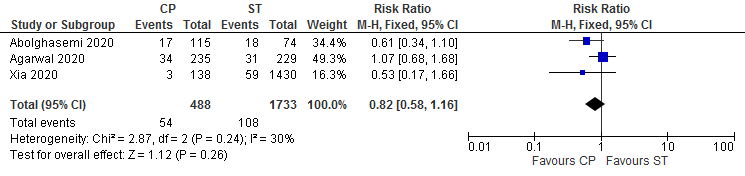

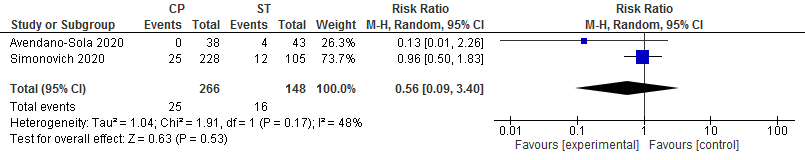


**Figure 2 -** **Forest plot assessing the mortality rate exclusively in the studies included in the length of hospitalization outcome’s meta-analysis.** **(A)** In the analysis of the mortality rate since hospital admission, the results showed a tendency for decreased mortality rates in the CP intervention group in comparison to standard treatment, although no statistical difference was obtained. The overall heterogenicity levels are low. **(B)** In the analysis of the mortality rate since CP treatment beginning, the results also showed a tendency for decreased mortality rates in the CP intervention group. The overall heterogeneity levels are moderated.

Different sizes of data markers correspond to the relative weight assigned in the pooled analysis. Diamond marker indicates the overall result.
